# Burden of malignant neoplasm of bone and articular cartilage from 1990 to 2021 and its predictions to 2030 in China compared with world

**DOI:** 10.3389/fonc.2025.1629679

**Published:** 2025-08-21

**Authors:** Cheng Chen, Xu Zheng, Yi Zhang, ZhenDong Li, Bing Li, YunFeng Yang, WeiBin Zhang

**Affiliations:** ^1^ Department of Orthopedics, Shanghai Sixth People’s Hospital Affiliated to Shanghai Jiao Tong University School of Medicine, Shanghai, China; ^2^ Department of Bone and Joint Surgery, Department of Orthopedics, Renji Hospital, School of Medicine, Shanghai Jiao Tong University, Shanghai, China; ^3^ Department of Orthopedics, Tongji Hospital, School of Medicine, Tongji University, Shanghai, China; ^4^ Department of Orthopaedics, Ruijin Hospital, Shanghai JiaoTong University School of Medicine, Shanghai, China

**Keywords:** malignant neoplasm of bone and articular cartilage, China, epidemiology, incidence, mortality, GBD 2021

## Abstract

**Objective:**

To comprehensively examine the incidence and mortality of malignant neoplasms of bone and articular cartilage (MNBAC) in China compared with the world, as well as its age-specific patterns and sex disparities.

**Methods:**

The MNBAC burden in China and the world was systematically assessed from 1990 to 2021 based on the Global Burden of Disease 2021, including incidence and mortality data. The estimated annual percentage change was calculated. The pattern of age and gender distribution was carried out. The future trends of MNBAC incidence and mortality through 2030 were predicted using the Bayesian age-period-cohort model.

**Results:**

From 1990 to 2021, China experienced rising trends in both the incidence and mortality of MNBAC, increasing at a much faster pace than the world. Before 2005, crude incidence and mortality rates in China increased but plateaued thereafter. In contrast, age-standardized rates displayed a decreasing trend from 2005 onward. During this period, the world experienced relatively stable incidence and mortality rates. The incidence and mortality rates of MNBAC increase with age. The MNBAC burden is higher in males. The rise in incidence and mortality rate in China was predominantly in the elderly. The burden prediction for MNBAC to 2030 showed that China would experience a more significant drop in incidence and mortality rates for MNBAC, along with a decrease in the incidence and mortality cases.

**Conclusions:**

From 1990 to 2021, the MNBAC burden in China remains heavy, with incidence and mortality increasing much faster than in the world. The projection results indicate a promising outlook for the future, which is encouraging news. Men and the elderly should be the key target for the public health policies of MNBAC.

## Introduction

Malignant neoplasm of bone and articular cartilage (MNBAC), including osteosarcoma, Ewing sarcoma, and chondrosarcoma, accounts for less than 1% of newly diagnosed malignant tumors (1). However, they pose a significant threat to health and well-being. For most countries, osteosarcoma accounts for 20-40% of all primary bone cancers, while Ewing sarcoma represents less than 20% (2). The proportion of chondrosarcoma varies considerably, ranging from <10% to over 45% (2). The overall age-standardized rate for bone cancer is 0.8–1.2/100000 in males and 0.5–1.0/100000 in females (2). The etiology of bone cancer remains unknown, but several risk factors have been identified by researchers, such as exposure to skeletal radiation and alkylating agents (3).

MNBAC presents significant challenges to healthcare systems worldwide due to their aggressive nature, rapid progression, and poor prognosis (4). The slow progress in treating MNBAC compared to other tumors is notable. The management of these tumors is costly, involving procedures such as surgery, chemotherapy, and radiation therapy (5–7). For localized diseases, surgical resection is the primary treatment option, often with adjuvant or neoadjuvant chemotherapy. However, over half of the patients with localized bone cancer experience recurrence, often with distant metastases (8). Besides, traditional chemotherapy is often accompanied by drug resistance and can cause severe systemic toxicity from several chemotherapeutic agents. Patients confront a myriad of challenges, including physical, emotional, and economic difficulties (5). Despite recent advancements in medical technology, a considerable number of patients still rapidly succumb to the disease (9, 10). Several factors may impede improvements in survival rates for MNBAC. The aggressive nature of these tumors limits the time available for patients to explore diverse treatment options, potentially leading to missed opportunities for personalized treatment plans. In the domain of basic research, the number of studies on MNBAC is lower compared to other types of malignancies. This disparity has resulted in a limited understanding of the pathogenesis, progression, and metastasis of these tumors, which, in turn, may impede the development of effective targeted therapies (11, 12). The stagnation in survival rate improvements underscores the need for enhanced research efforts and a deeper understanding of this disease. These challenges often translate into significant economic burdens on healthcare systems and substantial losses in societal productivity (13). Therefore, addressing these challenges requires significant investment in healthcare resources at the societal level.

Research indicates that the global burden of MNBAC has continuously increased from 1990 to 2021, especially in East Asia (14). At the country level, China had the highest incidence and mortality number in 2021, but there is a lack of detailed data in China (14). Previous epidemiological studies on the burden of MNBAC in China are outdated and not compared globally (15, 16). Given the social development and potential epidemiological changes, an updated comprehensive analysis of the burden of MNBAC in China is warranted. Besides, comparing with global data is helpful to gain a better understanding of the epidemiological patterns in China.

The Global Burden of Disease (GBD) study is a comprehensive and systematic global health assessment that aims to quantify the burden of disease, injury, and risk factors across the world. The GBD 2021 study builds upon and extends the GBD 2019, with notable updates in data sources and methodologies, thereby contributing significantly to a deeper understanding of global health dynamics (17–19). This study aims to provide a comprehensive examination of the incidence and mortality of MNBAC in China based on GBD 2021, in comparison with the world. By filling these knowledge gaps, this study aims to contribute to a better understanding of the impact of MNBAC on health in China and inform targeted public health strategies.

## Methods

The GBD 2021 encompasses over 371 diseases and injuries, along with 88 risk factors, and covers 204 countries and territories (17–19). The extensive assessment serves as a critical tool for identifying emerging health trends, monitoring progress over time, and informing evidence-based health policies and interventions. By revealing disparities in health outcomes across regions, GBD 2021 aids policymakers in prioritizing resources and designing targeted strategies to address the most pressing health challenges. The transparency and rigorous data analysis in GBD 2021 ensures the robustness and reliability of its findings, making it a pivotal resource for researchers, public health practitioners, and policymakers alike. Its contributions to our understanding of global health dynamics are invaluable in shaping a more equitable and sustainable future for global health. Population-related data was sourced from GBD (https://ghdx.healthdata.org/). Data on MNBAC in China and the world, spanning from 1990 to 2021, were extracted from the GBD 2021 dataset (https://vizhub.healthdata.org/gbd-results/). The 95% uncertainty interval (UI) was calculated from the 2.5th and 97.5th percentiles of the sampled outcomes.

### Statistical analysis

The burden of MNBAC in China was systematically assessed, alongside the global levels within the GBD 2021 framework. Incidence, mortality, and years of life lost (YLLs) of MNBAC were obtained from the GBD 2021 repository (https://vizhub.healthdata.org/gbd-results/). YLLs is a metric used in epidemiology and public health to quantify premature mortality or the number of years of potential life lost due to a particular cause of death. It is a measure that helps to understand the impact of premature deaths on a population by taking into account not only the number of deaths but also the age at which those deaths occur.

Estimated Annual Percentage Change (EAPC) is a statistical measure used to quantify the average rate of change in a particular variable over a specified period. The calculation of EAPC involves fitting a regression model to the logarithm of the rates, which allows for the estimation of the average annual change. To quantify the variation trend from 1990 to 2021, we calculated the EAPC with 95% confidence intervals (CI), with positive EAPC indicating an upward trajectory and negative EAPC suggesting a decline (20). Next, the pattern of age and gender distribution linked to MNBAC was carried out.

Using the Bayesian age-period-cohort model (21), we further predicted the future trends of MNBAC incidence and mortality rates and cases through 2030. The Bayesian age-period-cohort model is a sophisticated statistical framework used to analyze and interpret temporal trends in epidemiological and demographic data. This model is particularly useful for disentangling the effects of age, period, and cohort on observed rates, such as incidence or mortality rates, which are often interrelated and challenging to separate using traditional methods. To ensure smooth transitions across age, period, and cohort categories, second-order random walk priors were applied to these components. Posterior inference was executed utilizing Markov Chain Monte Carlo, which facilitated the derivation of credible intervals for the estimated effects. Statistical significance was determined at the threshold of P < 0.05. Statistical analysis and data visualization were executed using the R software (version 4.2.2).

## Results

From 1990 to 2021, the incidence number of MNBAC in China significantly increased from 6382 (95% UI: 4178 to 11228) in 1990 to 25937 (95% UI: 16243 to 34274) in 2021(**Table 1**). By contrast, the global incidence number of MNBAC significantly increased from 46583 (95% UI: 42151 to 54215) in 1990 to 91375 (95% UI: 73780 to 102470) in 2021. The crude incidence rate of MNBAC in China in 1990 was 0.54 (95% UI: 0.36 to 0.95), lower than that in the world [0.87 (95% UI: 0.79 to 1.02)]. However, the crude incidence rate of MNBAC in China in 2021 [1.82 (95% UI: 1.14 to 2.41)] was higher than that in the world [1.16 (95% UI: 0.93 to 1.3)]. A similar situation exists with the age-standardized incidence rate (ASIR). The ASIR of MNBAC in China in 1990 was 0.65 (95% UI: 0.42 to 1.15), lower than that in the world [0.97 (95% UI: 0.89 to 1.13)]. However, The ASIR of MNBAC in China in 2021 [1.42 (95% UI: 0.9 to 1.86)] was higher than that in the world [1.11(95% UI: 0.9 to 1.25)].

**Table 1:** The burden of MNBAC in 1990 and 2021 for both sexes in the world and China, with EAPC from 1990 and 2021.

Measure	Location	Number			CR, per 100k			ASR, per 100k		
		Number in 1990 (95% UI)	Number in 2021 (95% UI)	Number change rate (95% UI)	CR in 1990 (95% UI)	CR in 2021 (95% UI)	EAPC of CR, % per year (95% CI)	ASR in 1990 (95% UI)	ASR in 2021 (95% UI)	EAPC of ASR, % per year (95% CI)
Incidence	World	46583 (42151 to 54215)	91375 (73780 to 102470)	0.96 (0.61 to 1.23)	0.87 (0.79 to 1.02)	1.16 (0.93 to 1.3)	1.06 (0.98 to 1.13)	0.97 (0.89 to 1.13)	1.11 (0.9 to 1.25)	0.59 (0.51 to 0.68)
	China	6382 (4178 to 11228)	25938 (16243 to 34274)	3.06 (0.81 to 6.73)	0.54 (0.36 to 0.95)	1.82 (1.14 to 2.41)	4.84 (4.26 to 5.43)	0.65 (0.42 to 1.15)	1.42 (0.9 to 1.86)	3.37 (2.75 to 3.99)
Mortality	World	35552 (32334 to 42011)	66114 (53305 to 74467)	0.86 (0.53 to 1.12)	0.67 (0.61 to 0.79)	0.84 (0.68 to 0.94)	0.85 (0.77 to 0.93)	0.79 (0.73 to 0.93)	0.79 (0.64 to 0.89)	0.11 (0.02 to 0.21)
	China	5292 (3393 to 9393)	18085 (11288 to 24126)	2.42 (0.51 to 5.56)	0.45 (0.29 to 0.8)	1.27 (0.79 to 1.7)	4.19 (3.56 to 4.82)	0.58 (0.38 to 1.03)	0.93 (0.58 to 1.23)	2.21 (1.53 to 2.89)

MNBAC, Malignant neoplasm of bone and articular cartilage; CR, crude rate; ASR, age-standardized rate; EAPC, estimated annual percentage change; UI, uncertainty interval; CI, confidence interval.

With regards to mortality data of MNBAC, from 1990 to 2021, the mortality number of MNBAC in China significantly rose from 5292 (95% UI: 3393 to 9393) in 1990 to 18085 (95% UI: 11288 to 24126) in 2021. On the other hand, the mortality number of MNBAC globally rose from 35552 (95% UI: 32334 to 42011) in 1990 to 66114 (95% UI: 53305 to 74467) in 2021. The crude mortality rate of MNBAC in China in 1990 was 0.45 (95% UI: 0.29 to 0.8), lower than that in the world [0.67 (95% UI: 0.61 to 0.79)]. However, the crude mortality rate of MNBAC in China in 2021 [1.27 (95% UI: 0.79 to 1.7)] was higher than that in the world [0.84 (95% UI: 0.68 to 0.94)]. The age-standardized death rate (ASDR) of MNBAC in China rose from 0.58 (95% UI: 0.38 to 1.03) in 1990 to 0.93 (95% UI: 0.58 to 1.23) in 2021. The global ASDR of MNBAC remained relatively stable with a value of 0.79.

**Figure 1:**
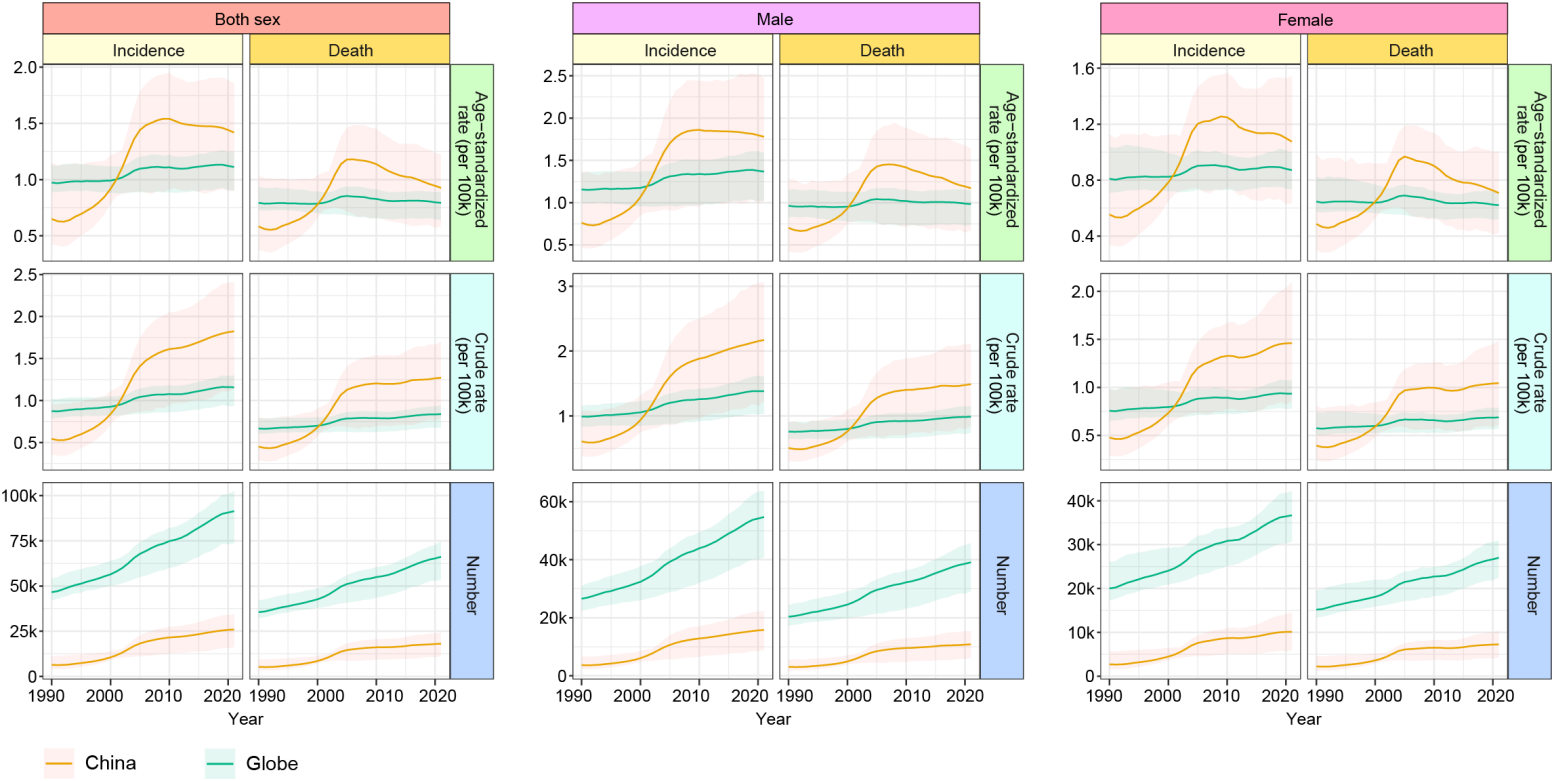
Epidemiological trends of MNBAC across gender in China and the world from 1990 to 2021. MNBAC, Malignant neoplasm of bone and articular cartilage.

**Figure 2:**
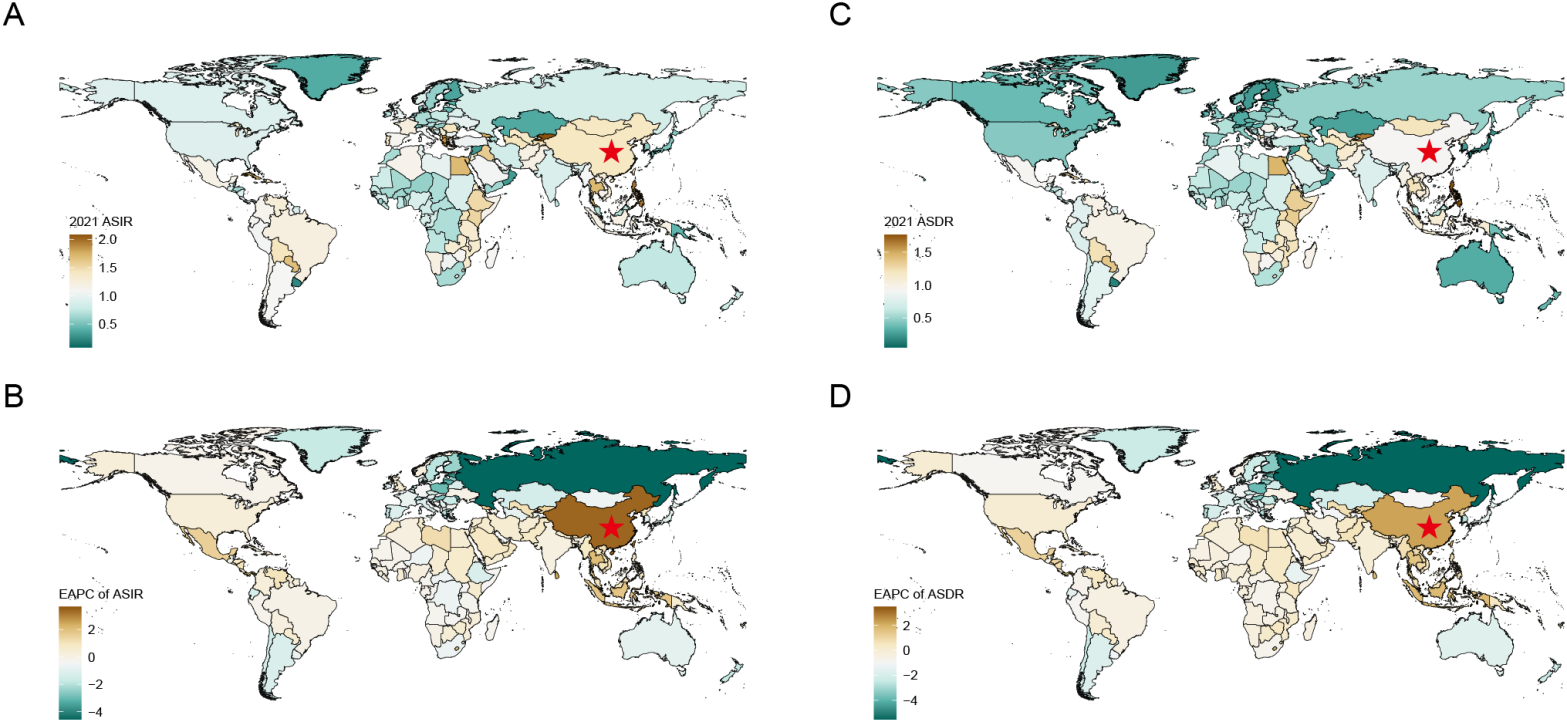
ASIR **(A)** and ASDR **(C)** maps of MNBAC by countries and territories in 2021. EPAC maps of ASIR **(B)** and ASDR **(D)** of MNBAC by countries and territories from 1990 to 2021. The red star marks China. MNBAC, Malignant neoplasm of bone and articular cartilage; ASIR, age-standardized incidence rate; ASDR, age-standardized death rate; EAPC, estimated annual percentage change.

**Figure 1** visualizes the temporal trends in the epidemiology of MNBAC between China and the world from 1990 to 2021. Both the incidence and mortality numbers in China and the world exhibit increasing trends. In China, the crude incidence and mortality rates of MNBAC increased before 2005 but plateaued afterward, while the age-standardized rates displayed a decreasing trend after 2005. In contrast, the world experienced relatively stable changes in incidence and mortality rates. Additionally, it is noteworthy that the incidence and mortality numbers and rates for males were higher than those for females, evident in both China and the world. The geographical distribution of MNBAC burden across countries and territories is presented in **Supplementary Tables S1**, **S2** and visualized in **Figure 2**. The age-standardized rate of MNBAC in China and EAPC are comparatively higher than those of other countries and territories.

**Figure 3:**
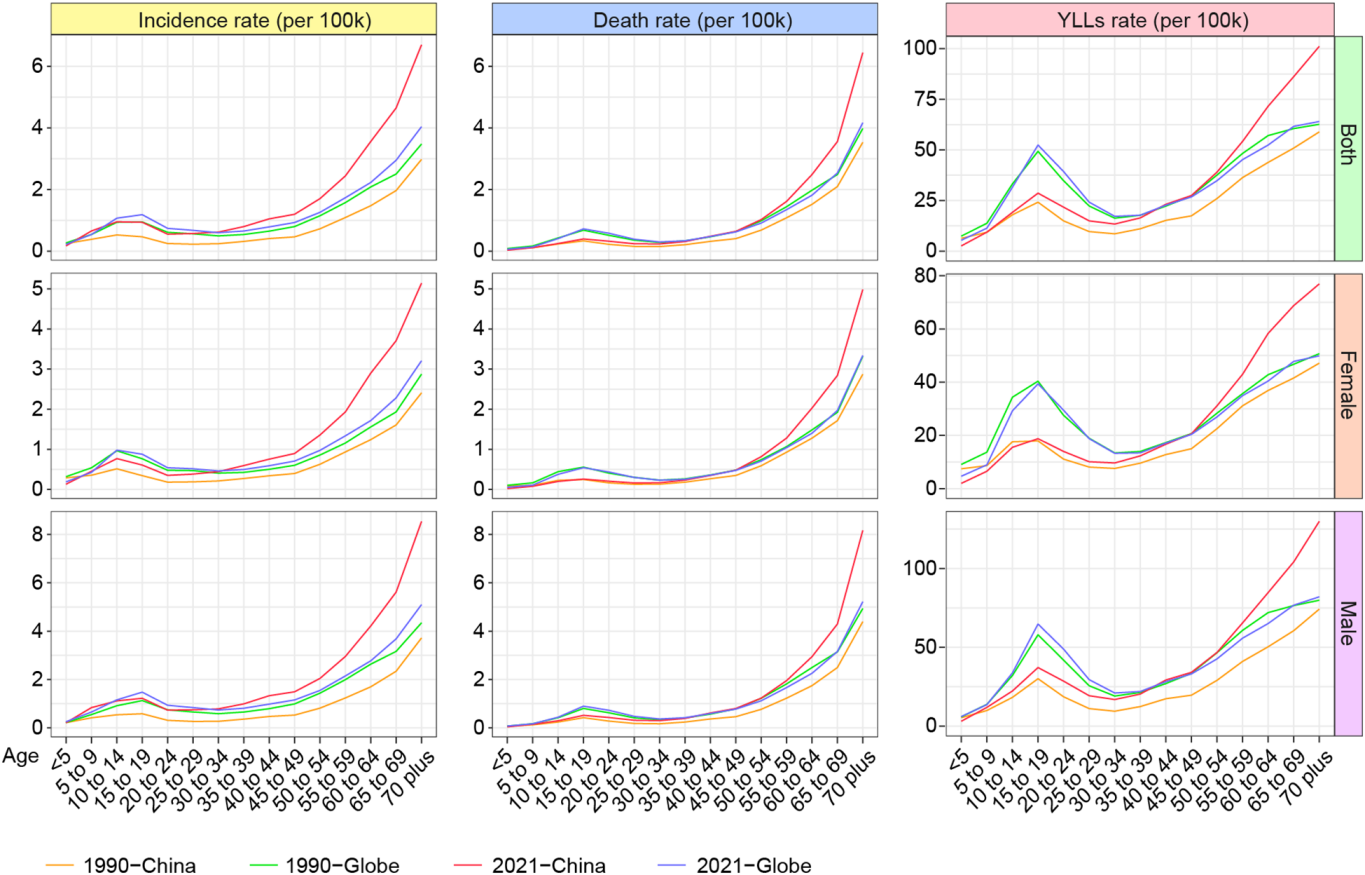
The incidence, mortality and YLLs rates of MNBAC in China and the world across age and gender in 1990 and 2021. MNBAC, Malignant neoplasm of bone and articular cartilage; YLLs, years of life lost.

Overall, the incidence and mortality rates of MNBAC increase with age (**Figure 3**). Notably, a small peak occurs during adolescence. It is important to note that death at younger ages results in a greater loss of potential life years compared to death at older ages. Therefore, YLLs were further used to compare the disease burden across ages. The analysis revealed a distinct bimodal distribution of YLLs rates, with peaks corresponding to adolescents and the elderly. Although the patterns of MNBAC burden by age are similar between males and females, the burden is higher in males. From 1990 to 2021, the incidence rate of MNBAC in various age groups in China increased, most significantly in the elderly. The rise in mortality rate in China was mainly reflected in the elderly, while the middle-aged and younger adults, as well as children, showed minimal changes. YLLs of MNBAC in adolescents in China had consistently been lower than in the world, but the YLLs of MNBAC in the elderly in China had increased over 30 years and was higher than in the world in 2021. In contrast, the global burden across different age groups changed little over 30 years.

**Figure 4:**
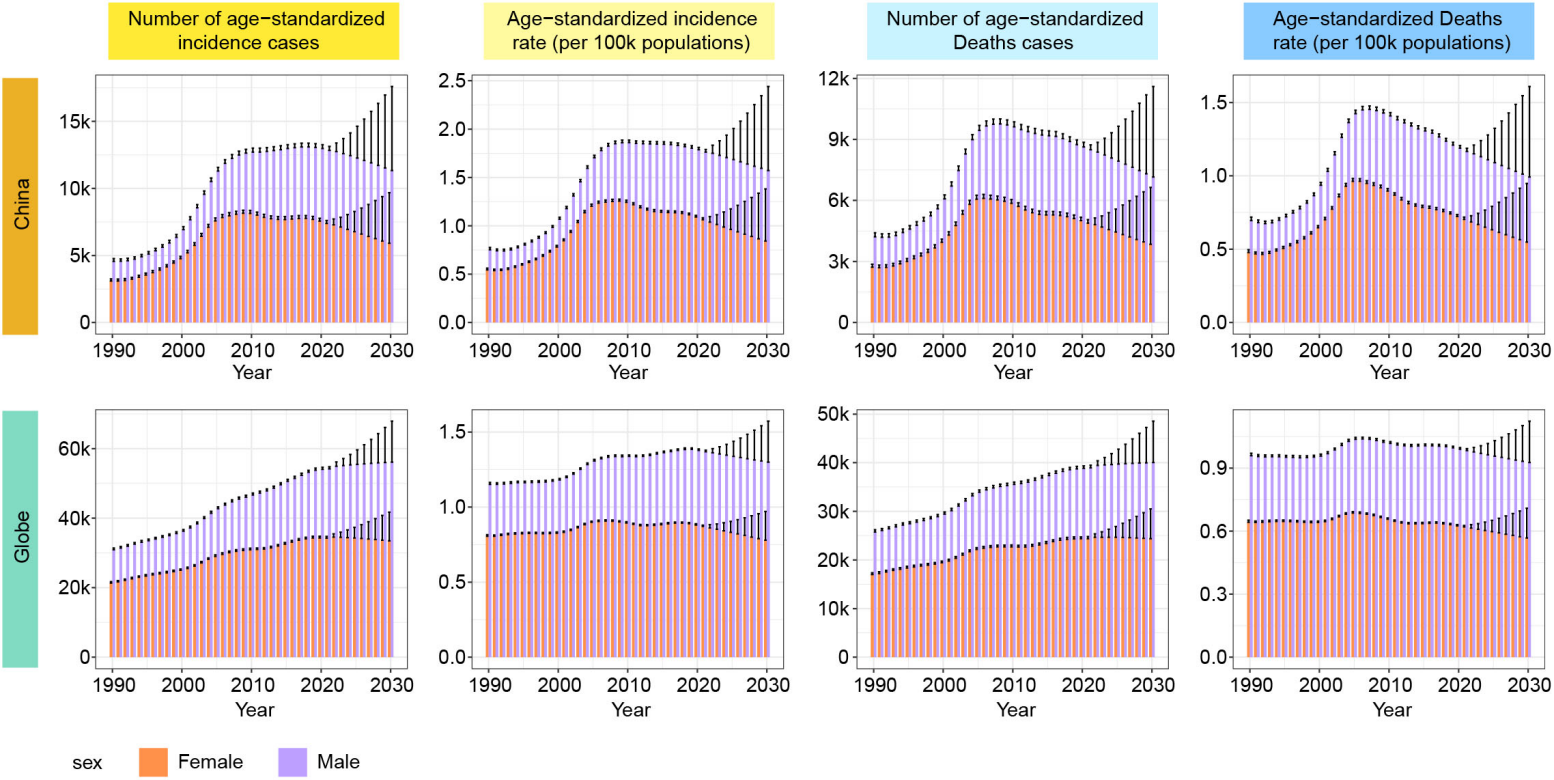
Prediction to 2030 of MNBAC in age-standardized rate and number of incidence and mortality in China and the world. MNBAC, Malignant neoplasm of bone and articular cartilage.

The burden prediction for MNBAC in China and globally up to 2030 was shown in **Figure 4**. Globally, the incidence rate of MNBAC for males was projected to reach 1.30 by 2030, with 56131 cases, while for females, the incidence rate was expected to be 0.78, resulting in 33499 cases. The mortality rates for global MNBAC in 2030 were estimated to be 0.93 for males, with 40037 cases, and 0.57 for females, resulting in 24394 cases. A slight decline in incidence and mortality rates for global MNBAC was predicted, with the incidence and mortality cases entering a plateau phase. In contrast, China would experience a more significant drop in incidence and mortality rates for MNBAC, along with a decrease in the incidence and mortality cases. By 2030, the incidence rate of MNBAC in Chinese males was projected to be 1.57, with 11331 cases, while for Chinese females, the incidence rate was predicted to be 0.84, resulting in 5909 cases. The mortality rates for MNBAC in 2030 were estimated to be 0.99 for Chinese males, with 7153 cases, and 0.55 for Chinese females, resulting in 3845 cases.

## Discussion

This study complements existing literature by providing a comprehensive analysis of the MNBAC burden in China over the past three decades and comparing it with the world within the same database source. The findings offer valuable insights for health policies regarding MNBAC in China. This study underscores the significant burden of MNBAC in China, with incidence and mortality increasing much faster than that in the world. The results also highlight the higher burden of MNBAC in males and the increase in incidence and mortality with age. The fastest-growing population for MNBAC incidence and mortality in China from 1990 to 2021 was the elderly, warranting special attention. The varying age and sex dynamics of the MNBAC burden emphasize the need for targeted interventions and age-specific strategies. Despite China’s heavy burden of MNBAC in the past three decades, the projection of improvement is encouraging news.

According to data from 368 registries in the National Central Cancer Registry of China, in 2015, it was estimated that there were 24200 new cases in China, with an incidence rate of 1.77 per 100000 population and an age-standardized incidence rate by world standard population of 1.32 per 100000. Regarding mortality rates, it was estimated that there were 17900 deaths in 2015, with a crude mortality rate of 1.31 per 100000 and an age-standardized mortality rate by the world standard population of 0.89 per 100000 (16). China still faces a significant burden of MNBAC, which is associated with its large population and the increasing incidence and mortality rates. The growth in incidence and mortality can be attributed to various factors, including changing demographic structures, environmental pollution, and unhealthy lifestyles, which pose ongoing challenges.

This study found that 2005 marked a significant turning point in the incidence and mortality of MNBAC in China. The reforms around 2005 should be understood as the initiation of a transformative process that gradually enhanced the health system’s capacity to respond to and manage the burden of diseases like bone tumors. The improvements in health system indicators provide strong evidence for the reforms’ positive impact on bone tumor outcomes. These reforms include the initiation of hospital-based health technology assessment in 2005 which provide the evidence needed to make clinical decisions at the administrative level (22). The period beginning in 2005 marked the steepest climb in health expenditure totals for both urban and rural demographics (23).

Before 2005, the incidence and mortality of MNBAC in China were on an upward trend, but this trend was curbed after 2005, which may be attributed to the following reasons: 1. Allocation of medical resources and healthcare system reform: 2005 marked a significant point in the reform of China’s healthcare system. Before this, China had experimented with market-oriented approaches in healthcare, but as issues such as significantly increased medical expenses and compromised public welfare became apparent, the government began to strengthen macroeconomic regulation and returned to a system primarily based on macroeconomic control in healthcare (24). This shift may have led to a more rational allocation and management of diagnostic and treatment resources for MNBAC, thereby influencing the incidence and mortality rates (25). 2. Cancer screening and early diagnosis: Post-2005, the Chinese government released a series of health plans to promote cancer prevention and control (26, 27). China initiated various targeted cancer screening programs, including early diagnosis and treatment projects for cancer in high-incidence rural and urban areas. These plans provided a roadmap and policy options for cancer control, enhanced cancer surveillance networks, and offered crucial information for policy formulation and evaluation. These measures may contribute to enhancing the prevention and treatment outcomes of MNBAC, thereby influencing their incidence and mortality rates. 3. Advancements in treatment technology: there were significant advancements in medical technology in China in the early 2000, particularly in the field of surgical techniques. With the development of medical technology, the introduction of advanced diagnostic and treatment concepts, especially progress in surgery, chemotherapy, and radiotherapy, improved the survival rates and quality of life for MNBAC patients. These technological advancements may help control the progression of the disease, thus reducing the incidence and mortality rates.

China bears a relatively higher burden of MNBAC compared to other regions of the world, particularly developed countries (28). These differences may be attributed to various factors: for instance, the uneven distribution of medical resources between urban and rural areas in China may lead to delayed diagnosis and treatment for patients in rural areas, impacting survival rates. Additionally, there may be disparities in surgical techniques and the concept of adjunctive therapies between China and developed countries, directly affecting treatment outcomes and survival rates for patients (26). Variances in economic status and health awareness may result in inadequacies in disease prevention, early diagnosis, and treatment for Chinese patients. Genetic susceptibility differences among different races and regions could also influence the incidence and survival outcomes of MNBAC (15, 16, 29). The combination of these factors may contribute to differences in the incidence, treatment outcomes, and patient survival rates of MNBAC between China and other regions worldwide. To improve survival rates, it is necessary to enhance the equitable distribution of medical resources, advance diagnostic and treatment technologies, raise public health awareness, and enhance cancer registration and monitoring systems.

This study found a higher disease burden of MNBAC in males compared to females. Differences in the burden of MNBAC between males and females have been observed in the previous study (2). The overall ASR of bone cancer is 0.8–1.2 per 100000 in males and 0.5–1.0 per 100000 in females (2). This disparity may be attributed to the potential influence of endogenous sex hormones (30, 31). Studies have suggested the presence of estrogen receptors in osteosarcoma cells, which may influence their survival (32). The increasing use of oral contraceptives and hormone replacement therapy may impact the epidemiological characteristics of MNBAC in female (33).

Adolescents and the elderly are key populations that require special attention, as revealed by this study. This is consistent with the age distribution of MNBAC in China reported in the literature (15). Osteosarcoma exhibits a higher incidence rate in adolescents, with an annual rate of 0.8 to 1.1 cases per 100000 individuals in the 15–19 age group; Notably, there is a significant second peak in incidence among elderly individuals (4, 34). Adolescents comprise one of the most financially affected age groups by MNBAC. This demographic tends to have lower rates of health insurance coverage, which can adversely affect health outcomes owing to reduced access to necessary tests and optimal treatment options (35, 36). The lack of adequate insurance is associated with lower survival rates, particularly in the advanced stages of MNBAC (36–38).

### Limitations of this study

Despite the methodological advancements and expanded data sources compared to GBD 2019, there are still limitations to the GBD 2021. This study is based on GBD 2021, therefore inheriting the same limitations. First, although GBD 2021 employs rigorously calibrated and validated models and algorithms for estimation, these methods rely on assumptions and simplifications that may not fully capture the complexity of real-world scenarios. Second, the study lacks subtype analysis for MNBAC. Third, there may be significant variations among different provinces in China, but due to data availability limitations, this study could not conduct a more detailed analysis of the epidemiological patterns at the provincial level.

## Conclusion

From 1990 to 2021, the MNBAC burden in China remains heavy, with incidence and mortality increasing much faster than in the world. The projection results indicate a promising outlook for the future, which is encouraging news. Men and the elderly should be the key target for the public health policies of MNBAC.

## Data Availability

Publicly available datasets were analyzed in this study. This data can be found here: https://vizhub.healthdata.org/gbd-results.

## Supplementary material

Supplementary Table 2 Mortality of malignant neoplasm of bone and articular cartilage in 1990 and 2021 for both sexes in 204 countries and territories, with EAPC from 1990 and 2021.

Supplementary Table 3 Prediction to 2030 of malignant neoplasm of bone and articular cartilage in age-standardized rate and number of incidence and mortality in China and the world.
