# Supplementary material for: Burden of malignant neoplasm of bone and articular cartilage from 1990 to 2021 and its predictions to 2030 in China compared with world
Source: Front Oncol. 2025 Aug 21;15:1629679. doi: 10.3389/fonc.2025.1629679 (PMC12408263; doi:10.3389/fonc.2025.1629679)
Supplement: Supplementary Table 2 — Mortality of malignant neoplasm of bone and articular cartilage in 1990 and 2021 for both sexes in 204 countries and territories, with EAPC from 1990 and 2021. [file Table1.docx]

**Burden of Malignant neoplasm of bone and articular cartilage from 1990 to 2021 and its predictions to 2030 in China compared with World**

**Supplementary Material**

**Contents**

**Table S1………………………………………………………………………………………. 2**

**Table S2……………………………………………………………………………….……… 23**

Table S1. Incidence of malignant neoplasm of bone and articular cartilage in 1990 and 2021 for both sexes in countries and territories, with EAPC from 1990 to 2021.

| **Location** | **Number** | | | **CR, per 100k** | | | **ASR, per 100k** | | |
| --- | --- | --- | --- | --- | --- | --- | --- | --- | --- |
|  | **Number in 1990 (95% UI)** | **Number in 2021 (95% UI)** | **Number change rate (95% UI)** | **CR in 1990 (95% UI)** | **CR in 2021 (95% UI)** | **EAPC of CR, % per year (95% CI)** | **ASR in 1990 (95% UI)** | **ASR in 2021 (95% UI)** | **EAPC of ASR, % per year (95% CI)** |
| Afghanistan | 94 (52 to 151) | 264 (150 to 409) | 1.82 (0.98 to 3.06) | 0.94 (0.52 to 1.52) | 0.84 (0.48 to 1.31) | -0.07 (-0.4 to 0.27) | 1.1 (0.66 to 1.72) | 1.21 (0.74 to 1.79) | 0.47 (0.31 to 0.62) |
| Albania | 52 (39 to 68) | 54 (28 to 91) | 0.03 (-0.45 to 0.58) | 1.58 (1.19 to 2.06) | 2.02 (1.05 to 3.43) | 0.94 (0.67 to 1.21) | 1.87 (1.39 to 2.48) | 1.54 (0.81 to 2.56) | -0.44 (-0.72 to -0.16) |
| Algeria | 248 (169 to 332) | 458 (327 to 647) | 0.84 (0.17 to 2.01) | 0.98 (0.67 to 1.31) | 1.04 (0.74 to 1.46) | 0.07 (-0.04 to 0.18) | 1.13 (0.8 to 1.43) | 1.15 (0.83 to 1.64) | 0.13 (0.02 to 0.23) |
| American Samoa | 0 (0 to 0) | 1 (0 to 1) | 0.97 (0.22 to 2.3) | 0.66 (0.46 to 0.95) | 1.26 (0.91 to 1.7) | 2.61 (2.34 to 2.88) | 0.86 (0.61 to 1.22) | 1.25 (0.9 to 1.69) | 1.7 (1.41 to 1.99) |
| Andorra | 0 (0 to 0) | 0 (0 to 0) | 0.39 (-0.22 to 1.44) | 0.17 (0.06 to 0.33) | 0.15 (0.06 to 0.28) | -0.35 (-0.52 to -0.18) | 0.17 (0.06 to 0.32) | 0.12 (0.04 to 0.22) | -1.02 (-1.16 to -0.89) |
| Angola | 53 (35 to 77) | 161 (100 to 252) | 2.02 (0.81 to 3.92) | 0.52 (0.34 to 0.75) | 0.49 (0.31 to 0.77) | -0.13 (-0.29 to 0.02) | 0.79 (0.51 to 1.09) | 0.73 (0.47 to 1.08) | -0.26 (-0.38 to -0.15) |
| Antigua and Barbuda | 0 (0 to 0) | 1 (1 to 1) | 0.74 (0.54 to 0.99) | 0.56 (0.49 to 0.61) | 0.65 (0.61 to 0.69) | 0.87 (0.58 to 1.16) | 0.57 (0.51 to 0.63) | 0.6 (0.57 to 0.64) | 0.5 (0.28 to 0.73) |
| Argentina | 546 (476 to 612) | 544 (499 to 597) | 0 (-0.12 to 0.13) | 1.65 (1.44 to 1.85) | 1.2 (1.1 to 1.31) | -1.01 (-1.23 to -0.79) | 1.66 (1.45 to 1.86) | 1.1 (1 to 1.2) | -1.26 (-1.49 to -1.04) |
| Armenia | 31 (22 to 41) | 38 (28 to 52) | 0.22 (-0.25 to 0.92) | 0.9 (0.64 to 1.2) | 1.26 (0.92 to 1.72) | 1.47 (1.23 to 1.71) | 1 (0.72 to 1.34) | 0.99 (0.72 to 1.36) | 0.37 (0.13 to 0.62) |
| Australia | 172 (162 to 183) | 218 (195 to 241) | 0.27 (0.13 to 0.42) | 1.02 (0.96 to 1.09) | 0.85 (0.76 to 0.94) | -0.65 (-0.93 to -0.38) | 0.97 (0.91 to 1.04) | 0.74 (0.66 to 0.83) | -0.98 (-1.3 to -0.65) |
| Austria | 72 (66 to 77) | 89 (79 to 100) | 0.24 (0.1 to 0.4) | 0.92 (0.85 to 0.99) | 0.99 (0.88 to 1.12) | -0.2 (-0.48 to 0.08) | 0.8 (0.74 to 0.86) | 0.72 (0.64 to 0.82) | -0.83 (-1.11 to -0.55) |
| Azerbaijan | 47 (27 to 78) | 71 (44 to 118) | 0.54 (-0.16 to 1.66) | 0.63 (0.37 to 1.07) | 0.68 (0.42 to 1.12) | 0.2 (0.1 to 0.29) | 0.73 (0.43 to 1.23) | 0.67 (0.42 to 1.11) | -0.24 (-0.32 to -0.15) |
| Bahamas | 2 (1 to 2) | 3 (2 to 3) | 0.67 (0.32 to 1.17) | 0.64 (0.58 to 0.72) | 0.71 (0.57 to 0.88) | 0.38 (0.13 to 0.62) | 0.76 (0.69 to 0.84) | 0.68 (0.55 to 0.84) | -0.33 (-0.57 to -0.08) |
| Bahrain | 4 (3 to 5) | 16 (12 to 21) | 3.29 (2.12 to 4.98) | 0.73 (0.57 to 0.97) | 1.04 (0.76 to 1.35) | 1.14 (0.9 to 1.39) | 1.16 (0.9 to 1.5) | 1.33 (0.97 to 1.77) | 0.54 (0.36 to 0.73) |
| Bangladesh | 841 (591 to 1165) | 1654 (1127 to 2290) | 0.97 (0.24 to 1.94) | 0.77 (0.54 to 1.07) | 1 (0.68 to 1.39) | 0.99 (0.88 to 1.11) | 0.93 (0.66 to 1.25) | 1.02 (0.7 to 1.42) | 0.37 (0.3 to 0.45) |
| Barbados | 2 (2 to 3) | 3 (3 to 4) | 0.34 (0.04 to 0.65) | 0.96 (0.87 to 1.04) | 1.08 (0.86 to 1.31) | 0.79 (0.6 to 0.98) | 0.87 (0.8 to 0.95) | 0.8 (0.63 to 0.98) | 0.13 (-0.07 to 0.33) |
| Belarus | 149 (133 to 167) | 109 (80 to 137) | -0.27 (-0.46 to -0.07) | 1.43 (1.27 to 1.6) | 1.17 (0.86 to 1.47) | -0.82 (-0.91 to -0.73) | 1.3 (1.16 to 1.45) | 0.9 (0.64 to 1.16) | -1.3 (-1.39 to -1.2) |
| Belgium | 161 (149 to 173) | 156 (138 to 174) | -0.03 (-0.14 to 0.09) | 1.61 (1.49 to 1.74) | 1.36 (1.2 to 1.51) | -0.81 (-1.18 to -0.44) | 1.39 (1.28 to 1.51) | 1.01 (0.89 to 1.14) | -1.42 (-1.81 to -1.02) |
| Belize | 1 (1 to 1) | 3 (2 to 3) | 1.15 (0.8 to 1.57) | 0.7 (0.59 to 0.79) | 0.65 (0.58 to 0.73) | 0.14 (-0.09 to 0.37) | 0.82 (0.66 to 0.95) | 0.74 (0.66 to 0.82) | -0.04 (-0.32 to 0.24) |
| Benin | 31 (22 to 44) | 85 (59 to 120) | 1.76 (0.86 to 3.21) | 0.64 (0.46 to 0.9) | 0.63 (0.44 to 0.89) | -0.1 (-0.19 to -0.01) | 0.72 (0.53 to 0.99) | 0.71 (0.5 to 0.97) | -0.19 (-0.28 to -0.11) |
| Bermuda | 0 (0 to 0) | 1 (0 to 1) | 0.45 (0.15 to 0.89) | 0.67 (0.55 to 0.76) | 0.91 (0.76 to 1.11) | 1.07 (0.81 to 1.33) | 0.66 (0.54 to 0.74) | 0.56 (0.47 to 0.69) | -0.42 (-0.61 to -0.23) |
| Bhutan | 4 (2 to 6) | 8 (5 to 11) | 0.95 (0.13 to 2.42) | 0.63 (0.38 to 0.9) | 1.02 (0.69 to 1.47) | 1.49 (1.36 to 1.62) | 0.79 (0.54 to 1.1) | 1.07 (0.74 to 1.54) | 0.93 (0.86 to 1) |
| Bolivia (Plurinational State of) | 70 (50 to 95) | 153 (97 to 225) | 1.19 (0.31 to 2.58) | 1.09 (0.78 to 1.49) | 1.3 (0.82 to 1.91) | 0.51 (0.45 to 0.57) | 1.34 (1 to 1.78) | 1.43 (0.92 to 2.11) | 0.15 (0.1 to 0.19) |
| Bosnia and Herzegovina | 38 (21 to 61) | 37 (24 to 56) | -0.02 (-0.45 to 0.54) | 0.83 (0.46 to 1.36) | 1.11 (0.73 to 1.68) | 0.94 (0.76 to 1.11) | 0.86 (0.48 to 1.4) | 0.76 (0.51 to 1.16) | -0.26 (-0.41 to -0.11) |
| Botswana | 9 (6 to 12) | 20 (15 to 27) | 1.34 (0.51 to 2.59) | 0.66 (0.45 to 0.91) | 0.86 (0.61 to 1.13) | 0.95 (0.78 to 1.12) | 0.95 (0.65 to 1.3) | 1.03 (0.75 to 1.36) | 0.4 (0.25 to 0.54) |
| Brazil | 1604 (1520 to 1686) | 2881 (2736 to 3033) | 0.8 (0.7 to 0.9) | 1.08 (1.02 to 1.14) | 1.31 (1.24 to 1.38) | 0.82 (0.72 to 0.92) | 1.36 (1.29 to 1.42) | 1.23 (1.17 to 1.3) | -0.06 (-0.17 to 0.05) |
| Brunei Darussalam | 1 (1 to 1) | 2 (1 to 2) | 0.8 (0.21 to 1.62) | 0.34 (0.21 to 0.51) | 0.36 (0.23 to 0.51) | 0.51 (0.32 to 0.71) | 0.38 (0.25 to 0.57) | 0.37 (0.23 to 0.53) | 0.3 (0.11 to 0.49) |
| Bulgaria | 125 (103 to 147) | 107 (83 to 133) | -0.15 (-0.36 to 0.16) | 1.44 (1.19 to 1.69) | 1.57 (1.22 to 1.97) | 0.64 (0.29 to 0.98) | 1.23 (1.02 to 1.46) | 1.03 (0.79 to 1.3) | -0.25 (-0.6 to 0.09) |
| Burkina Faso | 56 (38 to 80) | 127 (85 to 176) | 1.25 (0.48 to 2.25) | 0.59 (0.4 to 0.84) | 0.56 (0.37 to 0.77) | -0.07 (-0.24 to 0.1) | 0.69 (0.47 to 1.01) | 0.63 (0.44 to 0.86) | -0.24 (-0.4 to -0.08) |
| Burundi | 56 (38 to 83) | 113 (65 to 187) | 1.04 (0.34 to 2.02) | 1 (0.68 to 1.49) | 0.86 (0.49 to 1.42) | -0.54 (-0.6 to -0.47) | 1.29 (0.91 to 1.9) | 1.11 (0.66 to 1.84) | -0.58 (-0.65 to -0.51) |
| Cabo Verde | 2 (1 to 2) | 3 (2 to 4) | 1.03 (0.31 to 1.96) | 0.44 (0.28 to 0.66) | 0.57 (0.37 to 0.79) | 0.82 (0.74 to 0.9) | 0.45 (0.29 to 0.67) | 0.6 (0.39 to 0.83) | 0.97 (0.87 to 1.07) |
| Cambodia | 59 (32 to 84) | 168 (71 to 273) | 1.83 (0.65 to 3.37) | 0.58 (0.31 to 0.81) | 0.99 (0.42 to 1.6) | 1.87 (1.82 to 1.91) | 0.86 (0.49 to 1.18) | 1.16 (0.5 to 1.82) | 1.02 (0.98 to 1.06) |
| Cameroon | 80 (57 to 108) | 232 (154 to 347) | 1.9 (0.94 to 3.2) | 0.77 (0.54 to 1.04) | 0.73 (0.49 to 1.09) | -0.07 (-0.16 to 0.02) | 0.91 (0.65 to 1.21) | 0.83 (0.56 to 1.21) | -0.25 (-0.32 to -0.17) |
| Canada | 268 (252 to 283) | 394 (351 to 434) | 0.47 (0.31 to 0.63) | 0.98 (0.92 to 1.04) | 1.05 (0.94 to 1.16) | 0.22 (0.01 to 0.42) | 0.95 (0.89 to 1) | 0.92 (0.82 to 1.02) | -0.15 (-0.4 to 0.09) |
| Central African Republic | 15 (10 to 21) | 26 (16 to 39) | 0.71 (0.13 to 1.39) | 0.55 (0.38 to 0.78) | 0.47 (0.29 to 0.71) | -0.61 (-0.72 to -0.51) | 0.83 (0.58 to 1.16) | 0.69 (0.42 to 1) | -0.69 (-0.75 to -0.64) |
| Chad | 31 (21 to 45) | 102 (65 to 147) | 2.29 (1.08 to 3.88) | 0.52 (0.35 to 0.74) | 0.58 (0.36 to 0.83) | 0.53 (0.45 to 0.61) | 0.59 (0.41 to 0.87) | 0.67 (0.43 to 0.95) | 0.49 (0.45 to 0.54) |
| Chile | 216 (195 to 239) | 246 (227 to 268) | 0.14 (0.03 to 0.28) | 1.63 (1.47 to 1.8) | 1.31 (1.21 to 1.43) | -0.63 (-0.87 to -0.4) | 1.8 (1.64 to 1.96) | 1.15 (1.05 to 1.25) | -1.41 (-1.65 to -1.18) |
| China | 6382 (4178 to 11228) | 25938 (16243 to 34274) | 3.06 (0.81 to 6.73) | 0.54 (0.36 to 0.95) | 1.82 (1.14 to 2.41) | 4.84 (4.26 to 5.43) | 0.65 (0.42 to 1.15) | 1.42 (0.9 to 1.86) | 3.37 (2.75 to 3.99) |
| Colombia | 277 (256 to 300) | 586 (497 to 694) | 1.11 (0.77 to 1.52) | 0.85 (0.79 to 0.92) | 1.19 (1.01 to 1.41) | 1.2 (1.02 to 1.38) | 1.05 (0.98 to 1.12) | 1.15 (0.97 to 1.35) | 0.3 (0.12 to 0.48) |
| Comoros | 5 (3 to 7) | 10 (6 to 18) | 0.94 (0.11 to 2.61) | 1.09 (0.74 to 1.54) | 1.31 (0.75 to 2.38) | 0.1 (-0.34 to 0.54) | 1.38 (0.95 to 1.95) | 1.45 (0.84 to 2.63) | -0.25 (-0.57 to 0.07) |
| Congo | 15 (11 to 21) | 36 (24 to 56) | 1.41 (0.62 to 2.54) | 0.62 (0.44 to 0.86) | 0.66 (0.45 to 1.04) | 0.09 (-0.05 to 0.23) | 0.92 (0.66 to 1.23) | 0.87 (0.61 to 1.34) | -0.27 (-0.38 to -0.15) |
| Cook Islands | 0 (0 to 0) | 0 (0 to 0) | 0.41 (-0.05 to 1.06) | 0.25 (0.15 to 0.39) | 0.38 (0.21 to 0.58) | 1.14 (0.99 to 1.29) | 0.29 (0.17 to 0.42) | 0.33 (0.18 to 0.52) | 0.2 (0.04 to 0.35) |
| Costa Rica | 21 (20 to 24) | 50 (44 to 57) | 1.35 (0.99 to 1.75) | 0.71 (0.64 to 0.78) | 1.06 (0.92 to 1.21) | 1.39 (1 to 1.79) | 0.82 (0.75 to 0.89) | 0.98 (0.85 to 1.12) | 0.68 (0.35 to 1.01) |
| Côte d'Ivoire | 63 (44 to 90) | 164 (109 to 229) | 1.58 (0.77 to 2.65) | 0.52 (0.36 to 0.74) | 0.59 (0.39 to 0.82) | 0.52 (0.39 to 0.66) | 0.62 (0.42 to 0.85) | 0.65 (0.44 to 0.9) | 0.24 (0.14 to 0.33) |
| Croatia | 82 (72 to 93) | 86 (72 to 101) | 0.05 (-0.14 to 0.23) | 1.69 (1.48 to 1.92) | 2.04 (1.72 to 2.39) | 0.27 (-0.15 to 0.7) | 1.52 (1.33 to 1.73) | 1.24 (1.04 to 1.48) | -0.9 (-1.3 to -0.5) |
| Cuba | 164 (150 to 179) | 279 (239 to 319) | 0.7 (0.45 to 0.99) | 1.51 (1.38 to 1.65) | 2.47 (2.12 to 2.83) | 1.46 (1.27 to 1.64) | 1.51 (1.39 to 1.65) | 1.76 (1.52 to 2.01) | 0.28 (0.1 to 0.47) |
| Cyprus | 8 (6 to 12) | 15 (11 to 21) | 0.86 (0.31 to 1.92) | 1.05 (0.71 to 1.49) | 1.13 (0.81 to 1.51) | 0.56 (0.31 to 0.82) | 1.07 (0.72 to 1.48) | 0.93 (0.67 to 1.23) | 0.02 (-0.25 to 0.29) |
| Czechia | 122 (111 to 135) | 102 (85 to 123) | -0.17 (-0.33 to 0.05) | 1.19 (1.08 to 1.31) | 0.96 (0.8 to 1.16) | -0.53 (-0.86 to -0.19) | 1.02 (0.92 to 1.13) | 0.63 (0.52 to 0.78) | -1.3 (-1.66 to -0.94) |
| Democratic People's Republic of Korea | 180 (101 to 273) | 381 (179 to 612) | 1.12 (0.35 to 2.34) | 0.87 (0.49 to 1.33) | 1.45 (0.68 to 2.32) | 1.81 (1.69 to 1.93) | 1 (0.57 to 1.5) | 1.27 (0.6 to 2.02) | 0.95 (0.83 to 1.07) |
| Democratic Republic of the Congo | 207 (144 to 287) | 421 (253 to 634) | 1.04 (0.21 to 1.95) | 0.54 (0.38 to 0.75) | 0.47 (0.28 to 0.7) | -0.54 (-0.74 to -0.34) | 0.82 (0.57 to 1.1) | 0.68 (0.41 to 0.99) | -0.69 (-0.84 to -0.54) |
| Denmark | 43 (40 to 46) | 45 (39 to 50) | 0.05 (-0.07 to 0.19) | 0.83 (0.77 to 0.89) | 0.76 (0.67 to 0.85) | -0.88 (-1.23 to -0.54) | 0.69 (0.64 to 0.74) | 0.52 (0.46 to 0.58) | -1.59 (-1.98 to -1.19) |
| Djibouti | 4 (2 to 6) | 14 (8 to 26) | 2.88 (1.2 to 5.19) | 0.9 (0.53 to 1.4) | 1.15 (0.61 to 2.09) | 0.72 (0.52 to 0.93) | 1.19 (0.73 to 1.84) | 1.4 (0.77 to 2.52) | 0.49 (0.33 to 0.65) |
| Dominica | 1 (0 to 1) | 1 (1 to 1) | 0.48 (0.03 to 1.23) | 0.83 (0.59 to 1.14) | 1.33 (1 to 1.7) | 1.71 (1.55 to 1.87) | 0.9 (0.65 to 1.24) | 1.21 (0.91 to 1.57) | 1.15 (0.98 to 1.33) |
| Dominican Republic | 68 (46 to 101) | 146 (100 to 211) | 1.14 (0.51 to 2.28) | 0.96 (0.65 to 1.41) | 1.33 (0.91 to 1.92) | 1.32 (1.17 to 1.47) | 1.18 (0.78 to 1.73) | 1.39 (0.96 to 2.01) | 0.79 (0.64 to 0.94) |
| Ecuador | 145 (123 to 169) | 195 (158 to 236) | 0.34 (0.04 to 0.75) | 1.46 (1.24 to 1.69) | 1.08 (0.87 to 1.3) | -0.8 (-0.98 to -0.63) | 1.77 (1.53 to 2.05) | 1.13 (0.92 to 1.36) | -1.3 (-1.47 to -1.13) |
| Egypt | 605 (459 to 806) | 1377 (980 to 2026) | 1.28 (0.57 to 2.24) | 1.09 (0.83 to 1.46) | 1.3 (0.93 to 1.92) | 0.49 (0.31 to 0.66) | 1.5 (1.11 to 1.98) | 1.7 (1.21 to 2.5) | 0.46 (0.29 to 0.62) |
| El Salvador | 24 (19 to 29) | 51 (32 to 71) | 1.08 (0.45 to 1.91) | 0.46 (0.35 to 0.55) | 0.78 (0.49 to 1.09) | 1.99 (1.88 to 2.1) | 0.54 (0.42 to 0.64) | 0.79 (0.5 to 1.1) | 1.35 (1.27 to 1.43) |
| Equatorial Guinea | 2 (1 to 3) | 9 (5 to 16) | 2.97 (1.4 to 6.21) | 0.55 (0.35 to 0.79) | 0.61 (0.36 to 1.03) | 0.41 (0.29 to 0.52) | 0.8 (0.52 to 1.13) | 0.85 (0.52 to 1.33) | 0.28 (0.19 to 0.36) |
| Eritrea | 32 (21 to 48) | 70 (38 to 122) | 1.2 (0.32 to 2.12) | 0.93 (0.62 to 1.41) | 1.06 (0.57 to 1.85) | 0.25 (0.18 to 0.32) | 1.28 (0.9 to 1.87) | 1.33 (0.75 to 2.27) | -0.03 (-0.09 to 0.03) |
| Estonia | 16 (14 to 19) | 8 (7 to 10) | -0.48 (-0.59 to -0.34) | 1.03 (0.89 to 1.18) | 0.64 (0.53 to 0.76) | -2.7 (-3.24 to -2.15) | 0.94 (0.81 to 1.07) | 0.41 (0.33 to 0.48) | -3.77 (-4.29 to -3.24) |
| Eswatini | 6 (4 to 8) | 12 (7 to 17) | 0.94 (0.33 to 1.87) | 0.75 (0.52 to 0.99) | 1.01 (0.63 to 1.5) | 1.05 (0.75 to 1.35) | 1.18 (0.86 to 1.56) | 1.35 (0.84 to 2.02) | 0.52 (0.18 to 0.86) |
| Ethiopia | 732 (543 to 1007) | 1329 (941 to 1962) | 0.82 (0.24 to 1.85) | 1.45 (1.07 to 1.99) | 1.22 (0.86 to 1.8) | -0.94 (-1.15 to -0.74) | 1.96 (1.48 to 2.62) | 1.51 (1.1 to 2.17) | -1.26 (-1.43 to -1.08) |
| Fiji | 5 (3 to 7) | 9 (6 to 12) | 0.74 (0.2 to 1.77) | 0.65 (0.41 to 0.98) | 0.93 (0.63 to 1.34) | 1.1 (0.8 to 1.41) | 0.79 (0.51 to 1.19) | 0.99 (0.68 to 1.43) | 0.66 (0.37 to 0.95) |
| Finland | 42 (38 to 45) | 27 (24 to 31) | -0.34 (-0.43 to -0.25) | 0.83 (0.77 to 0.9) | 0.49 (0.44 to 0.56) | -1.8 (-1.98 to -1.63) | 0.71 (0.66 to 0.77) | 0.36 (0.32 to 0.4) | -2.43 (-2.62 to -2.24) |
| France | 1059 (976 to 1119) | 1116 (986 to 1244) | 0.05 (-0.05 to 0.17) | 1.83 (1.69 to 1.94) | 1.68 (1.49 to 1.87) | -0.28 (-0.42 to -0.14) | 1.59 (1.47 to 1.68) | 1.25 (1.12 to 1.4) | -0.77 (-0.89 to -0.64) |
| Gabon | 6 (5 to 8) | 13 (8 to 20) | 0.93 (0.22 to 1.99) | 0.66 (0.48 to 0.85) | 0.69 (0.46 to 1.08) | -0.03 (-0.11 to 0.05) | 0.87 (0.63 to 1.14) | 0.87 (0.58 to 1.33) | -0.19 (-0.31 to -0.06) |
| Gambia | 6 (4 to 8) | 20 (13 to 30) | 2.19 (1.05 to 3.74) | 0.64 (0.45 to 0.86) | 0.84 (0.54 to 1.25) | 0.67 (0.41 to 0.92) | 0.74 (0.53 to 0.98) | 0.92 (0.62 to 1.35) | 0.48 (0.25 to 0.71) |
| Georgia | 68 (48 to 89) | 84 (66 to 108) | 0.25 (-0.16 to 0.89) | 1.23 (0.87 to 1.61) | 2.34 (1.83 to 2.99) | 2.2 (1.64 to 2.77) | 1.15 (0.81 to 1.52) | 1.77 (1.41 to 2.22) | 1.53 (0.99 to 2.07) |
| Germany | 595 (541 to 655) | 748 (678 to 813) | 0.26 (0.12 to 0.4) | 0.74 (0.68 to 0.82) | 0.88 (0.79 to 0.95) | 0.17 (-0.01 to 0.36) | 0.64 (0.58 to 0.7) | 0.66 (0.61 to 0.72) | -0.45 (-0.74 to -0.16) |
| Ghana | 88 (60 to 132) | 229 (151 to 332) | 1.6 (0.66 to 3.21) | 0.59 (0.4 to 0.88) | 0.67 (0.44 to 0.97) | 0.41 (0.34 to 0.48) | 0.67 (0.46 to 1.01) | 0.75 (0.51 to 1.06) | 0.36 (0.3 to 0.42) |
| Greece | 389 (367 to 410) | 274 (252 to 295) | -0.3 (-0.35 to -0.25) | 3.74 (3.54 to 3.94) | 2.69 (2.48 to 2.9) | -1.67 (-1.98 to -1.37) | 3.05 (2.9 to 3.22) | 1.87 (1.73 to 2.02) | -2.07 (-2.34 to -1.79) |
| Greenland | 0 (0 to 1) | 0 (0 to 0) | -0.36 (-0.66 to 0.31) | 0.64 (0.32 to 0.99) | 0.4 (0.28 to 0.57) | -1.17 (-1.6 to -0.75) | 0.72 (0.37 to 1.12) | 0.39 (0.27 to 0.56) | -1.79 (-2.23 to -1.35) |
| Grenada | 1 (1 to 1) | 1 (1 to 1) | 0.04 (-0.15 to 0.3) | 0.98 (0.83 to 1.15) | 0.87 (0.75 to 0.98) | 0.29 (-0.03 to 0.61) | 1.06 (0.9 to 1.22) | 0.81 (0.71 to 0.91) | -0.25 (-0.66 to 0.16) |
| Guam | 0 (0 to 1) | 1 (0 to 1) | 0.42 (-0.2 to 0.92) | 0.26 (0.18 to 0.37) | 0.32 (0.19 to 0.45) | 1.36 (1.04 to 1.68) | 0.32 (0.21 to 0.45) | 0.29 (0.17 to 0.41) | 0.34 (0.04 to 0.65) |
| Guatemala | 65 (54 to 78) | 124 (107 to 145) | 0.9 (0.5 to 1.42) | 0.78 (0.65 to 0.93) | 0.79 (0.68 to 0.92) | 0.6 (0.38 to 0.82) | 1.11 (0.95 to 1.22) | 0.9 (0.78 to 1.05) | -0.3 (-0.49 to -0.12) |
| Guinea | 49 (35 to 67) | 108 (74 to 155) | 1.19 (0.47 to 2.46) | 0.82 (0.58 to 1.12) | 0.8 (0.55 to 1.16) | -0.03 (-0.1 to 0.03) | 0.86 (0.64 to 1.12) | 0.88 (0.62 to 1.24) | 0.06 (0.01 to 0.12) |
| Guinea-Bissau | 7 (5 to 10) | 13 (8 to 18) | 0.82 (0.23 to 1.68) | 0.71 (0.46 to 1.03) | 0.63 (0.4 to 0.89) | -0.35 (-0.45 to -0.25) | 0.84 (0.56 to 1.21) | 0.75 (0.51 to 1.03) | -0.36 (-0.43 to -0.29) |
| Guyana | 7 (6 to 8) | 8 (6 to 10) | 0.13 (-0.16 to 0.46) | 0.89 (0.76 to 1.02) | 1.03 (0.81 to 1.31) | 1.1 (0.85 to 1.34) | 1.16 (1.02 to 1.31) | 1.09 (0.87 to 1.38) | 0.25 (0.05 to 0.46) |
| Haiti | 45 (27 to 71) | 88 (51 to 138) | 0.94 (0.25 to 2.23) | 0.71 (0.42 to 1.11) | 0.68 (0.4 to 1.07) | 0.13 (0.05 to 0.2) | 0.93 (0.58 to 1.35) | 0.87 (0.51 to 1.36) | -0.02 (-0.08 to 0.05) |
| Honduras | 18 (12 to 26) | 51 (28 to 82) | 1.83 (0.85 to 3.45) | 0.38 (0.25 to 0.54) | 0.51 (0.28 to 0.81) | 0.81 (0.68 to 0.95) | 0.49 (0.33 to 0.67) | 0.64 (0.35 to 1) | 0.85 (0.69 to 1.01) |
| Hungary | 156 (143 to 171) | 82 (68 to 98) | -0.47 (-0.57 to -0.36) | 1.5 (1.38 to 1.65) | 0.86 (0.71 to 1.02) | -2.12 (-2.59 to -1.66) | 1.24 (1.14 to 1.37) | 0.59 (0.48 to 0.7) | -2.73 (-3.21 to -2.24) |
| Iceland | 3 (3 to 3) | 5 (4 to 5) | 0.73 (0.49 to 1.01) | 1.09 (1 to 1.2) | 1.37 (1.19 to 1.56) | -0.25 (-0.77 to 0.28) | 1.04 (0.96 to 1.14) | 1.12 (0.98 to 1.28) | -0.84 (-1.37 to -0.3) |
| India | 6079 (4380 to 7309) | 12201 (9929 to 15376) | 1.01 (0.62 to 1.76) | 0.71 (0.51 to 0.86) | 0.86 (0.7 to 1.09) | 0.53 (0.43 to 0.64) | 0.84 (0.61 to 0.99) | 0.89 (0.73 to 1.12) | 0.11 (0.01 to 0.2) |
| Indonesia | 1019 (591 to 1307) | 3155 (1413 to 4909) | 2.1 (0.95 to 3.33) | 0.55 (0.32 to 0.71) | 1.13 (0.51 to 1.76) | 2.39 (2.29 to 2.5) | 0.71 (0.43 to 0.9) | 1.2 (0.54 to 1.83) | 1.81 (1.66 to 1.96) |
| Iran (Islamic Republic of) | 370 (250 to 453) | 660 (494 to 786) | 0.79 (0.42 to 1.45) | 0.65 (0.44 to 0.79) | 0.77 (0.58 to 0.92) | 0.47 (0.32 to 0.62) | 0.75 (0.56 to 0.89) | 0.83 (0.63 to 0.99) | 0.5 (0.41 to 0.59) |
| Iraq | 206 (148 to 280) | 557 (414 to 762) | 1.71 (0.92 to 3.1) | 1.12 (0.8 to 1.52) | 1.35 (1.01 to 1.85) | 0.87 (0.76 to 0.98) | 1.42 (1.04 to 1.88) | 1.6 (1.19 to 2.17) | 0.6 (0.51 to 0.69) |
| Ireland | 47 (43 to 51) | 45 (40 to 50) | -0.04 (-0.17 to 0.1) | 1.3 (1.2 to 1.42) | 0.91 (0.8 to 1.02) | -0.87 (-1.19 to -0.55) | 1.21 (1.12 to 1.32) | 0.73 (0.64 to 0.82) | -1.18 (-1.45 to -0.91) |
| Israel | 46 (42 to 51) | 68 (60 to 75) | 0.45 (0.25 to 0.65) | 0.94 (0.84 to 1.03) | 0.7 (0.62 to 0.78) | -1.04 (-1.21 to -0.87) | 0.93 (0.83 to 1.03) | 0.64 (0.57 to 0.71) | -1.3 (-1.47 to -1.12) |
| Italy | 1180 (1122 to 1230) | 872 (783 to 943) | -0.26 (-0.32 to -0.2) | 2.08 (1.98 to 2.17) | 1.46 (1.31 to 1.58) | -0.78 (-1.16 to -0.4) | 1.74 (1.67 to 1.82) | 1.07 (0.97 to 1.16) | -1.17 (-1.54 to -0.81) |
| Jamaica | 27 (23 to 31) | 35 (26 to 45) | 0.26 (-0.09 to 0.6) | 1.16 (0.97 to 1.32) | 1.24 (0.94 to 1.62) | 0.27 (-0.06 to 0.59) | 1.26 (1.04 to 1.44) | 1.17 (0.89 to 1.53) | -0.19 (-0.5 to 0.11) |
| Japan | 918 (887 to 945) | 799 (736 to 841) | -0.13 (-0.18 to -0.09) | 0.73 (0.7 to 0.75) | 0.63 (0.58 to 0.66) | -0.38 (-0.59 to -0.18) | 0.72 (0.69 to 0.74) | 0.54 (0.51 to 0.56) | -0.93 (-1.1 to -0.76) |
| Jordan | 33 (25 to 44) | 144 (100 to 190) | 3.4 (1.93 to 5.29) | 0.87 (0.66 to 1.17) | 1.17 (0.81 to 1.54) | 1.01 (0.92 to 1.11) | 1.1 (0.83 to 1.47) | 1.33 (0.93 to 1.75) | 0.77 (0.66 to 0.88) |
| Kazakhstan | 80 (62 to 102) | 72 (52 to 97) | -0.11 (-0.44 to 0.36) | 0.49 (0.38 to 0.62) | 0.38 (0.27 to 0.51) | -1.13 (-1.48 to -0.77) | 0.55 (0.42 to 0.69) | 0.38 (0.28 to 0.51) | -1.43 (-1.75 to -1.1) |
| Kenya | 240 (182 to 304) | 646 (482 to 924) | 1.7 (0.99 to 2.79) | 1.04 (0.79 to 1.31) | 1.29 (0.96 to 1.85) | 0.86 (0.75 to 0.98) | 1.34 (1.01 to 1.69) | 1.54 (1.17 to 2.19) | 0.58 (0.51 to 0.65) |
| Kiribati | 0 (0 to 0) | 1 (0 to 1) | 1.21 (0.46 to 2.52) | 0.38 (0.25 to 0.53) | 0.52 (0.27 to 0.82) | 1.13 (0.87 to 1.39) | 0.47 (0.32 to 0.64) | 0.6 (0.32 to 0.93) | 0.87 (0.63 to 1.11) |
| Kuwait | 12 (10 to 13) | 33 (28 to 39) | 1.79 (1.28 to 2.45) | 0.69 (0.61 to 0.77) | 0.71 (0.59 to 0.85) | 0.55 (-0.83 to 1.95) | 0.9 (0.81 to 1) | 0.88 (0.73 to 1.05) | 0.6 (-0.78 to 2.01) |
| Kyrgyzstan | 56 (42 to 74) | 125 (91 to 169) | 1.23 (0.39 to 2.47) | 1.25 (0.95 to 1.66) | 1.82 (1.32 to 2.46) | 1.37 (0.89 to 1.87) | 1.42 (1.08 to 1.86) | 2.09 (1.52 to 2.79) | 1.41 (0.93 to 1.89) |
| Lao People's Democratic Republic | 26 (13 to 37) | 69 (30 to 118) | 1.67 (0.57 to 3.43) | 0.62 (0.32 to 0.9) | 0.93 (0.4 to 1.6) | 1.47 (1.41 to 1.54) | 0.88 (0.48 to 1.26) | 1.17 (0.51 to 1.99) | 0.97 (0.87 to 1.08) |
| Latvia | 36 (32 to 40) | 22 (18 to 26) | -0.4 (-0.53 to -0.25) | 1.36 (1.21 to 1.52) | 1.17 (0.97 to 1.4) | -1.41 (-1.86 to -0.95) | 1.23 (1.08 to 1.38) | 0.72 (0.6 to 0.87) | -2.56 (-3.01 to -2.11) |
| Lebanon | 26 (19 to 34) | 64 (47 to 90) | 1.48 (0.64 to 2.58) | 0.87 (0.64 to 1.15) | 1.16 (0.85 to 1.62) | 0.99 (0.77 to 1.22) | 0.98 (0.72 to 1.31) | 1.1 (0.8 to 1.53) | 0.43 (0.27 to 0.6) |
| Lesotho | 9 (6 to 11) | 18 (12 to 24) | 1 (0.3 to 2.05) | 0.57 (0.41 to 0.74) | 0.93 (0.66 to 1.3) | 2 (1.73 to 2.27) | 0.77 (0.55 to 1.02) | 1.21 (0.86 to 1.67) | 1.9 (1.61 to 2.18) |
| Liberia | 17 (12 to 23) | 37 (25 to 54) | 1.1 (0.41 to 2.07) | 0.71 (0.49 to 0.95) | 0.67 (0.45 to 0.98) | -0.01 (-0.37 to 0.34) | 0.81 (0.56 to 1.05) | 0.76 (0.52 to 1.1) | 0.07 (-0.2 to 0.35) |
| Libya | 26 (18 to 35) | 63 (44 to 91) | 1.45 (0.49 to 2.71) | 0.61 (0.44 to 0.83) | 0.91 (0.64 to 1.33) | 1.54 (1.42 to 1.66) | 0.75 (0.55 to 0.99) | 0.98 (0.69 to 1.4) | 1.22 (1.07 to 1.36) |
| Lithuania | 40 (36 to 45) | 20 (17 to 23) | -0.51 (-0.6 to -0.41) | 1.1 (0.99 to 1.22) | 0.73 (0.61 to 0.85) | -2.02 (-2.47 to -1.56) | 1.01 (0.92 to 1.13) | 0.46 (0.38 to 0.53) | -3.2 (-3.68 to -2.72) |
| Luxembourg | 5 (5 to 6) | 6 (5 to 6) | 0.08 (-0.03 to 0.21) | 1.4 (1.33 to 1.47) | 0.89 (0.81 to 0.99) | -1.64 (-1.78 to -1.49) | 1.29 (1.22 to 1.36) | 0.68 (0.62 to 0.76) | -2.35 (-2.51 to -2.19) |
| Madagascar | 108 (75 to 170) | 265 (146 to 464) | 1.44 (0.51 to 2.43) | 0.91 (0.63 to 1.43) | 0.93 (0.51 to 1.63) | -0.03 (-0.18 to 0.11) | 1.18 (0.84 to 1.79) | 1.11 (0.65 to 1.88) | -0.25 (-0.36 to -0.14) |
| Malawi | 96 (64 to 142) | 196 (114 to 335) | 1.04 (0.33 to 1.86) | 0.98 (0.65 to 1.45) | 1.01 (0.59 to 1.72) | 0.06 (-0.02 to 0.14) | 1.27 (0.89 to 1.82) | 1.23 (0.76 to 2.08) | -0.13 (-0.21 to -0.05) |
| Malaysia | 67 (37 to 98) | 215 (106 to 323) | 2.19 (1.41 to 3.5) | 0.38 (0.21 to 0.55) | 0.67 (0.33 to 1.02) | 1.91 (1.63 to 2.19) | 0.52 (0.29 to 0.74) | 0.7 (0.35 to 1.07) | 1 (0.8 to 1.2) |
| Maldives | 1 (1 to 2) | 3 (2 to 5) | 1.93 (0.96 to 3.25) | 0.52 (0.34 to 0.68) | 0.66 (0.45 to 0.88) | 0.81 (0.57 to 1.05) | 0.79 (0.53 to 1.02) | 0.77 (0.52 to 1.04) | -0.03 (-0.17 to 0.11) |
| Mali | 54 (37 to 76) | 136 (96 to 178) | 1.53 (0.73 to 2.68) | 0.62 (0.42 to 0.87) | 0.56 (0.4 to 0.74) | -0.18 (-0.25 to -0.1) | 0.7 (0.5 to 0.92) | 0.65 (0.47 to 0.84) | -0.19 (-0.25 to -0.13) |
| Malta | 5 (5 to 6) | 7 (6 to 8) | 0.33 (0.15 to 0.55) | 1.37 (1.24 to 1.5) | 1.52 (1.33 to 1.72) | 0.24 (-0.01 to 0.48) | 1.32 (1.19 to 1.44) | 1.21 (1.04 to 1.4) | -0.55 (-0.83 to -0.27) |
| Marshall Islands | 0 (0 to 0) | 0 (0 to 1) | 1.32 (0.41 to 2.62) | 0.28 (0.08 to 0.57) | 0.53 (0.14 to 1.04) | 1.88 (1.68 to 2.09) | 0.41 (0.13 to 0.84) | 0.61 (0.17 to 1.19) | 1.13 (0.92 to 1.34) |
| Mauritania | 14 (11 to 19) | 33 (21 to 49) | 1.27 (0.45 to 2.31) | 0.7 (0.52 to 0.93) | 0.74 (0.49 to 1.11) | -0.07 (-0.17 to 0.03) | 0.8 (0.59 to 1.03) | 0.81 (0.56 to 1.22) | -0.2 (-0.28 to -0.11) |
| Mauritius | 10 (10 to 11) | 20 (19 to 21) | 0.99 (0.83 to 1.15) | 0.93 (0.88 to 0.98) | 1.59 (1.47 to 1.68) | 3.11 (1.56 to 4.68) | 1.17 (1.11 to 1.23) | 1.22 (1.13 to 1.29) | 1.44 (-0.07 to 2.96) |
| Mexico | 565 (541 to 588) | 1521 (1370 to 1680) | 1.69 (1.42 to 1.98) | 0.66 (0.63 to 0.69) | 1.18 (1.06 to 1.3) | 2.41 (1.97 to 2.86) | 0.86 (0.84 to 0.89) | 1.18 (1.06 to 1.3) | 1.54 (1.08 to 2) |
| Micronesia (Federated States of) | 0 (0 to 1) | 1 (0 to 1) | 0.53 (-0.08 to 1.24) | 0.45 (0.18 to 0.8) | 0.7 (0.21 to 1.34) | 1.39 (1.29 to 1.48) | 0.6 (0.25 to 1.06) | 0.77 (0.24 to 1.44) | 0.76 (0.65 to 0.88) |
| Monaco | 0 (0 to 0) | 0 (0 to 0) | 0.33 (-0.09 to 0.99) | 0.22 (0.07 to 0.4) | 0.24 (0.09 to 0.41) | -0.16 (-0.3 to -0.01) | 0.18 (0.06 to 0.31) | 0.18 (0.06 to 0.3) | -0.47 (-0.65 to -0.29) |
| Mongolia | 27 (17 to 39) | 41 (30 to 53) | 0.52 (0.04 to 1.36) | 1.25 (0.78 to 1.82) | 1.23 (0.91 to 1.59) | 0.01 (-0.17 to 0.18) | 1.73 (1.12 to 2.51) | 1.42 (1.05 to 1.82) | -0.71 (-0.85 to -0.57) |
| Montenegro | 8 (4 to 11) | 7 (5 to 10) | -0.03 (-0.36 to 0.41) | 1.2 (0.67 to 1.73) | 1.18 (0.86 to 1.61) | 0.12 (0 to 0.24) | 1.19 (0.67 to 1.72) | 0.92 (0.67 to 1.26) | -0.67 (-0.81 to -0.53) |
| Morocco | 119 (78 to 165) | 239 (155 to 351) | 1.01 (0.26 to 2.08) | 0.47 (0.31 to 0.65) | 0.64 (0.42 to 0.94) | 1.02 (0.95 to 1.1) | 0.55 (0.38 to 0.75) | 0.66 (0.44 to 0.98) | 0.7 (0.63 to 0.76) |
| Mozambique | 137 (93 to 196) | 328 (189 to 544) | 1.4 (0.46 to 2.52) | 1.02 (0.7 to 1.47) | 1.06 (0.61 to 1.75) | 0.28 (0.17 to 0.38) | 1.31 (0.91 to 1.81) | 1.35 (0.8 to 2.24) | 0.27 (0.19 to 0.35) |
| Myanmar | 268 (144 to 405) | 574 (262 to 937) | 1.14 (0.23 to 2.59) | 0.66 (0.36 to 1) | 1.02 (0.47 to 1.66) | 1.31 (1.21 to 1.42) | 0.83 (0.47 to 1.22) | 1.09 (0.5 to 1.77) | 0.83 (0.7 to 0.96) |
| Namibia | 12 (9 to 14) | 24 (16 to 35) | 1.07 (0.32 to 2.12) | 0.82 (0.63 to 1.03) | 0.98 (0.65 to 1.45) | 0.41 (0.16 to 0.65) | 1.15 (0.89 to 1.44) | 1.21 (0.79 to 1.77) | -0.08 (-0.36 to 0.2) |
| Nauru | 0 (0 to 0) | 0 (0 to 0) | 0.48 (-0.06 to 1.18) | 0.48 (0.19 to 0.85) | 0.66 (0.19 to 1.19) | 0.89 (0.76 to 1.02) | 0.64 (0.26 to 1.09) | 0.81 (0.24 to 1.42) | 0.67 (0.56 to 0.79) |
| Nepal | 126 (88 to 170) | 269 (196 to 366) | 1.13 (0.42 to 2.12) | 0.65 (0.45 to 0.87) | 0.86 (0.63 to 1.17) | 1.11 (0.94 to 1.27) | 0.79 (0.56 to 1.04) | 0.91 (0.67 to 1.23) | 0.57 (0.41 to 0.73) |
| Netherlands | 135 (126 to 143) | 149 (131 to 165) | 0.1 (-0.03 to 0.23) | 0.9 (0.85 to 0.96) | 0.86 (0.76 to 0.96) | -0.47 (-0.68 to -0.26) | 0.83 (0.77 to 0.88) | 0.59 (0.53 to 0.66) | -1.48 (-1.73 to -1.22) |
| New Zealand | 49 (45 to 53) | 46 (42 to 51) | -0.06 (-0.17 to 0.07) | 1.44 (1.31 to 1.55) | 0.9 (0.81 to 0.99) | -1.54 (-2.04 to -1.03) | 1.38 (1.26 to 1.5) | 0.8 (0.73 to 0.89) | -1.83 (-2.34 to -1.31) |
| Nicaragua | 24 (18 to 32) | 55 (42 to 71) | 1.3 (0.73 to 2.34) | 0.62 (0.47 to 0.82) | 0.82 (0.63 to 1.06) | 1.31 (1.12 to 1.5) | 0.75 (0.58 to 1.02) | 0.92 (0.7 to 1.18) | 1.02 (0.81 to 1.24) |
| Niger | 50 (33 to 69) | 125 (81 to 186) | 1.5 (0.64 to 2.86) | 0.62 (0.42 to 0.86) | 0.5 (0.32 to 0.74) | -0.71 (-0.88 to -0.55) | 0.72 (0.51 to 0.99) | 0.59 (0.4 to 0.86) | -0.7 (-0.81 to -0.59) |
| Nigeria | 674 (492 to 887) | 1875 (1314 to 2538) | 1.78 (1.05 to 2.74) | 0.75 (0.55 to 0.99) | 0.81 (0.57 to 1.1) | 0.34 (0.19 to 0.48) | 0.84 (0.61 to 1.13) | 0.87 (0.63 to 1.16) | 0.14 (0.07 to 0.21) |
| Niue | 0 (0 to 0) | 0 (0 to 0) | 0.64 (0.07 to 1.49) | 0.52 (0.2 to 1.03) | 1.18 (0.4 to 2.34) | 1.72 (1.39 to 2.06) | 0.53 (0.2 to 1.04) | 1.14 (0.38 to 2.3) | 1.4 (1.02 to 1.78) |
| North Macedonia | 43 (34 to 53) | 52 (34 to 80) | 0.22 (-0.21 to 0.69) | 2.13 (1.72 to 2.65) | 2.38 (1.54 to 3.66) | 0.38 (0.14 to 0.62) | 2.19 (1.77 to 2.72) | 1.89 (1.23 to 2.92) | -0.43 (-0.68 to -0.18) |
| Northern Mariana Islands | 0 (0 to 0) | 0 (0 to 0) | 0.84 (0.09 to 2.12) | 0.33 (0.21 to 0.46) | 0.56 (0.37 to 0.74) | 2.35 (1.88 to 2.83) | 0.43 (0.3 to 0.6) | 0.52 (0.35 to 0.7) | 0.99 (0.52 to 1.46) |
| Norway | 32 (31 to 34) | 59 (54 to 64) | 0.85 (0.7 to 1) | 0.76 (0.72 to 0.79) | 1.1 (1 to 1.19) | 0.3 (-0.28 to 0.88) | 0.65 (0.62 to 0.68) | 0.93 (0.85 to 1.01) | 0.14 (-0.51 to 0.79) |
| Oman | 4 (2 to 5) | 10 (7 to 14) | 1.88 (0.72 to 3.67) | 0.18 (0.11 to 0.28) | 0.22 (0.15 to 0.29) | 0.87 (0.59 to 1.15) | 0.26 (0.15 to 0.4) | 0.32 (0.23 to 0.43) | 0.9 (0.69 to 1.12) |
| Pakistan | 840 (638 to 1081) | 2489 (1773 to 3552) | 1.96 (1.04 to 3.29) | 0.76 (0.57 to 0.97) | 1.06 (0.75 to 1.51) | 1.03 (0.93 to 1.14) | 0.91 (0.7 to 1.14) | 1.2 (0.86 to 1.68) | 0.83 (0.73 to 0.94) |
| Palau | 0 (0 to 0) | 0 (0 to 0) | 0.43 (-0.06 to 1.24) | 0.07 (0.03 to 0.13) | 0.09 (0.04 to 0.15) | 0.6 (0.43 to 0.77) | 0.09 (0.03 to 0.15) | 0.09 (0.04 to 0.15) | 0.15 (-0.02 to 0.32) |
| Palestine | 19 (12 to 28) | 62 (47 to 82) | 2.27 (1.38 to 4.17) | 0.93 (0.61 to 1.39) | 1.21 (0.92 to 1.59) | 1.19 (0.94 to 1.43) | 1.23 (0.82 to 1.77) | 1.5 (1.17 to 1.96) | 0.94 (0.76 to 1.13) |
| Panama | 10 (9 to 10) | 40 (33 to 48) | 3.21 (2.47 to 4.03) | 0.4 (0.37 to 0.43) | 0.94 (0.77 to 1.12) | 2.59 (2.23 to 2.94) | 0.46 (0.44 to 0.49) | 0.92 (0.76 to 1.11) | 2.04 (1.68 to 2.4) |
| Papua New Guinea | 11 (3 to 26) | 37 (7 to 85) | 2.22 (1.13 to 3.95) | 0.28 (0.06 to 0.64) | 0.35 (0.07 to 0.81) | 0.65 (0.49 to 0.8) | 0.36 (0.09 to 0.83) | 0.43 (0.09 to 1) | 0.54 (0.38 to 0.7) |
| Paraguay | 48 (36 to 63) | 110 (77 to 155) | 1.29 (0.54 to 2.26) | 1.19 (0.9 to 1.56) | 1.54 (1.08 to 2.16) | 0.97 (0.83 to 1.11) | 1.48 (1.12 to 1.91) | 1.67 (1.17 to 2.34) | 0.53 (0.38 to 0.68) |
| Peru | 216 (154 to 283) | 373 (269 to 511) | 0.73 (0.06 to 1.61) | 1 (0.71 to 1.31) | 1.03 (0.74 to 1.41) | 0.16 (0.03 to 0.3) | 1.11 (0.8 to 1.45) | 1.06 (0.76 to 1.45) | -0.19 (-0.33 to -0.04) |
| Philippines | 785 (673 to 1044) | 1921 (1519 to 2624) | 1.45 (1 to 2.07) | 1.25 (1.07 to 1.66) | 1.7 (1.34 to 2.32) | 1.21 (1.06 to 1.37) | 1.65 (1.41 to 2.18) | 1.96 (1.55 to 2.68) | 0.74 (0.54 to 0.95) |
| Poland | 734 (703 to 766) | 410 (374 to 446) | -0.44 (-0.5 to -0.39) | 1.92 (1.84 to 2.01) | 1.07 (0.98 to 1.17) | -2.13 (-2.42 to -1.84) | 1.8 (1.73 to 1.89) | 0.76 (0.7 to 0.83) | -3 (-3.3 to -2.71) |
| Portugal | 234 (218 to 250) | 201 (179 to 223) | -0.14 (-0.23 to -0.03) | 2.31 (2.15 to 2.47) | 1.9 (1.69 to 2.1) | -1.06 (-1.42 to -0.71) | 2.12 (1.97 to 2.28) | 1.39 (1.24 to 1.56) | -1.91 (-2.24 to -1.58) |
| Puerto Rico | 11 (9 to 12) | 32 (27 to 38) | 1.99 (1.43 to 2.66) | 0.3 (0.26 to 0.33) | 0.97 (0.8 to 1.15) | 4.11 (2.67 to 5.58) | 0.29 (0.26 to 0.33) | 0.62 (0.52 to 0.74) | 2.73 (1.24 to 4.25) |
| Qatar | 2 (1 to 2) | 13 (8 to 18) | 6.34 (4.07 to 9.35) | 0.39 (0.26 to 0.54) | 0.43 (0.29 to 0.61) | 0.78 (0.48 to 1.09) | 0.68 (0.47 to 0.9) | 0.75 (0.5 to 1.05) | 0.79 (0.55 to 1.03) |
| Republic of Korea | 310 (210 to 408) | 334 (256 to 441) | 0.08 (-0.28 to 0.79) | 0.7 (0.48 to 0.92) | 0.65 (0.5 to 0.85) | -0.41 (-0.52 to -0.3) | 0.73 (0.51 to 0.95) | 0.57 (0.44 to 0.75) | -1.07 (-1.21 to -0.93) |
| Republic of Moldova | 84 (73 to 93) | 35 (31 to 40) | -0.58 (-0.65 to -0.49) | 1.88 (1.65 to 2.1) | 0.98 (0.86 to 1.11) | -2.63 (-3.03 to -2.22) | 1.89 (1.66 to 2.11) | 0.69 (0.61 to 0.79) | -3.65 (-4.05 to -3.25) |
| Romania | 579 (478 to 687) | 404 (336 to 479) | -0.3 (-0.42 to -0.14) | 2.48 (2.04 to 2.94) | 2.13 (1.78 to 2.53) | -0.66 (-0.82 to -0.5) | 2.26 (1.88 to 2.66) | 1.43 (1.2 to 1.67) | -1.67 (-1.86 to -1.48) |
| Russian Federation | 4376 (4192 to 4527) | 1473 (1362 to 1577) | -0.66 (-0.69 to -0.64) | 2.9 (2.78 to 3) | 1.02 (0.94 to 1.09) | -4.37 (-4.71 to -4.03) | 2.67 (2.56 to 2.77) | 0.84 (0.78 to 0.9) | -4.58 (-4.91 to -4.25) |
| Rwanda | 87 (59 to 131) | 138 (75 to 253) | 0.59 (-0.08 to 1.55) | 1.21 (0.82 to 1.82) | 1.04 (0.57 to 1.91) | -0.75 (-0.9 to -0.6) | 1.56 (1.1 to 2.29) | 1.24 (0.7 to 2.22) | -1.07 (-1.22 to -0.92) |
| Saint Kitts and Nevis | 1 (0 to 1) | 1 (0 to 1) | 0.05 (-0.13 to 0.28) | 1.21 (1.07 to 1.33) | 0.89 (0.75 to 1.07) | -0.85 (-1.23 to -0.48) | 1.24 (1.11 to 1.35) | 0.88 (0.74 to 1.04) | -0.9 (-1.21 to -0.59) |
| Saint Lucia | 1 (1 to 1) | 1 (1 to 2) | 0.49 (0.18 to 0.81) | 0.65 (0.59 to 0.72) | 0.75 (0.61 to 0.89) | 0.49 (0.33 to 0.65) | 0.79 (0.72 to 0.86) | 0.65 (0.54 to 0.78) | -0.62 (-0.78 to -0.47) |
| Saint Vincent and the Grenadines | 1 (0 to 1) | 2 (2 to 3) | 3.87 (3.14 to 4.73) | 0.46 (0.42 to 0.51) | 2.17 (1.89 to 2.5) | 4.46 (3.18 to 5.75) | 0.53 (0.48 to 0.57) | 1.97 (1.72 to 2.28) | 3.67 (2.37 to 4.99) |
| Samoa | 2 (1 to 3) | 3 (2 to 5) | 0.6 (0.16 to 1.82) | 1.07 (0.57 to 1.88) | 1.35 (0.81 to 2.18) | 0.9 (0.75 to 1.06) | 1.34 (0.71 to 2.4) | 1.56 (0.95 to 2.52) | 0.61 (0.47 to 0.76) |
| San Marino | 0 (0 to 0) | 0 (0 to 1) | -0.02 (-0.39 to 0.43) | 1.43 (1.03 to 1.98) | 1.01 (0.63 to 1.6) | -0.44 (-0.74 to -0.14) | 1.25 (0.9 to 1.72) | 0.76 (0.48 to 1.17) | -0.94 (-1.21 to -0.68) |
| Sao Tome and Principe | 1 (1 to 1) | 2 (1 to 2) | 0.89 (0.08 to 2.16) | 0.67 (0.45 to 1.01) | 0.7 (0.44 to 1.08) | 0.16 (0 to 0.32) | 0.67 (0.46 to 1.01) | 0.76 (0.5 to 1.12) | 0.33 (0.23 to 0.44) |
| Saudi Arabia | 97 (64 to 133) | 335 (233 to 457) | 2.46 (1.28 to 4.39) | 0.61 (0.4 to 0.84) | 0.89 (0.62 to 1.21) | 1.31 (1.2 to 1.42) | 0.83 (0.57 to 1.14) | 1.06 (0.77 to 1.44) | 0.89 (0.8 to 0.98) |
| Senegal | 54 (38 to 76) | 113 (75 to 158) | 1.09 (0.33 to 1.97) | 0.71 (0.49 to 1) | 0.71 (0.47 to 1) | 0.15 (0 to 0.31) | 0.78 (0.56 to 1.1) | 0.78 (0.52 to 1.08) | 0.07 (-0.06 to 0.2) |
| Serbia | 175 (106 to 270) | 172 (127 to 227) | -0.02 (-0.44 to 0.58) | 1.82 (1.11 to 2.8) | 1.93 (1.42 to 2.54) | 0.15 (0.09 to 0.2) | 1.73 (1.04 to 2.68) | 1.3 (0.95 to 1.72) | -0.97 (-1.04 to -0.91) |
| Seychelles | 0 (0 to 0) | 0 (0 to 0) | 0.68 (0.27 to 1.29) | 0.2 (0.06 to 0.34) | 0.24 (0.06 to 0.4) | 0.95 (0.4 to 1.51) | 0.23 (0.07 to 0.38) | 0.21 (0.06 to 0.36) | 0.3 (-0.21 to 0.81) |
| Sierra Leone | 23 (15 to 34) | 50 (34 to 72) | 1.19 (0.48 to 2.22) | 0.55 (0.37 to 0.81) | 0.56 (0.38 to 0.82) | 0.17 (0.01 to 0.33) | 0.61 (0.41 to 0.91) | 0.63 (0.42 to 0.89) | 0.17 (0.04 to 0.3) |
| Singapore | 20 (19 to 22) | 29 (25 to 32) | 0.41 (0.23 to 0.61) | 0.67 (0.61 to 0.72) | 0.5 (0.44 to 0.56) | -1.24 (-1.56 to -0.91) | 0.72 (0.67 to 0.78) | 0.53 (0.47 to 0.61) | -1.37 (-1.75 to -0.99) |
| Slovakia | 63 (45 to 85) | 69 (50 to 94) | 0.11 (-0.26 to 0.56) | 1.18 (0.86 to 1.61) | 1.28 (0.93 to 1.74) | 0.21 (0.15 to 0.26) | 1.11 (0.8 to 1.52) | 0.93 (0.68 to 1.26) | -0.55 (-0.6 to -0.49) |
| Slovenia | 19 (17 to 20) | 17 (14 to 21) | -0.07 (-0.25 to 0.13) | 0.94 (0.85 to 1.03) | 0.83 (0.68 to 1) | -0.48 (-0.91 to -0.04) | 0.84 (0.76 to 0.91) | 0.5 (0.41 to 0.6) | -1.73 (-2.18 to -1.28) |
| Solomon Islands | 1 (0 to 2) | 3 (1 to 7) | 2.17 (1.19 to 3.93) | 0.29 (0.07 to 0.66) | 0.45 (0.11 to 0.95) | 1.31 (1.17 to 1.44) | 0.41 (0.1 to 0.93) | 0.56 (0.15 to 1.21) | 0.96 (0.83 to 1.08) |
| Somalia | 67 (43 to 106) | 170 (90 to 295) | 1.53 (0.52 to 2.68) | 0.85 (0.54 to 1.33) | 0.79 (0.42 to 1.36) | -0.37 (-0.52 to -0.22) | 1.3 (0.84 to 1.97) | 1.17 (0.66 to 1.95) | -0.35 (-0.43 to -0.27) |
| South Africa | 220 (145 to 278) | 344 (286 to 444) | 0.56 (0.2 to 1.31) | 0.59 (0.39 to 0.75) | 0.6 (0.5 to 0.78) | -0.29 (-0.56 to -0.02) | 0.74 (0.48 to 0.93) | 0.66 (0.55 to 0.84) | -0.72 (-0.98 to -0.45) |
| South Sudan | 55 (35 to 91) | 106 (55 to 199) | 0.95 (0.17 to 1.85) | 0.93 (0.6 to 1.56) | 1.1 (0.57 to 2.06) | 0.39 (0.1 to 0.69) | 1.26 (0.83 to 2.05) | 1.38 (0.78 to 2.54) | 0.21 (0.07 to 0.35) |
| Spain | 708 (667 to 743) | 648 (579 to 721) | -0.08 (-0.17 to 0.01) | 1.82 (1.72 to 1.92) | 1.42 (1.27 to 1.58) | -0.88 (-1.08 to -0.68) | 1.6 (1.51 to 1.69) | 1.12 (1.01 to 1.24) | -1.18 (-1.33 to -1.03) |
| Sri Lanka | 62 (49 to 83) | 186 (95 to 303) | 1.99 (0.42 to 4.24) | 0.36 (0.28 to 0.49) | 0.83 (0.43 to 1.36) | 3.59 (3.16 to 4.02) | 0.44 (0.35 to 0.59) | 0.74 (0.38 to 1.2) | 2.55 (2.1 to 3.01) |
| Sudan | 121 (66 to 190) | 329 (201 to 520) | 1.72 (0.64 to 3.46) | 0.6 (0.33 to 0.95) | 0.76 (0.46 to 1.2) | 0.82 (0.73 to 0.91) | 0.75 (0.45 to 1.09) | 0.89 (0.58 to 1.32) | 0.65 (0.57 to 0.72) |
| Suriname | 3 (2 to 4) | 5 (4 to 7) | 0.89 (0.26 to 1.84) | 0.73 (0.54 to 0.97) | 0.92 (0.66 to 1.26) | 0.87 (0.74 to 1) | 0.87 (0.64 to 1.14) | 0.88 (0.64 to 1.21) | 0.21 (0.12 to 0.3) |
| Sweden | 107 (99 to 114) | 73 (65 to 83) | -0.31 (-0.4 to -0.22) | 1.24 (1.15 to 1.33) | 0.71 (0.62 to 0.8) | -1.59 (-1.88 to -1.3) | 1.13 (1.04 to 1.21) | 0.59 (0.52 to 0.67) | -1.96 (-2.33 to -1.59) |
| Switzerland | 60 (56 to 64) | 59 (51 to 67) | -0.01 (-0.13 to 0.13) | 0.87 (0.81 to 0.94) | 0.66 (0.57 to 0.75) | -1.26 (-1.48 to -1.03) | 0.77 (0.71 to 0.83) | 0.49 (0.43 to 0.56) | -1.94 (-2.21 to -1.66) |
| Syrian Arab Republic | 27 (15 to 42) | 44 (24 to 71) | 0.64 (0.04 to 1.65) | 0.21 (0.11 to 0.33) | 0.31 (0.17 to 0.51) | 1.13 (0.94 to 1.33) | 0.26 (0.15 to 0.41) | 0.31 (0.17 to 0.49) | 0.49 (0.44 to 0.54) |
| Taiwan (Province of China) | 205 (192 to 218) | 226 (203 to 250) | 0.1 (-0.01 to 0.23) | 1.01 (0.94 to 1.07) | 0.96 (0.86 to 1.06) | 0.3 (-0.14 to 0.74) | 1.11 (1.04 to 1.18) | 0.69 (0.62 to 0.76) | -1.08 (-1.49 to -0.67) |
| Tajikistan | 67 (44 to 95) | 124 (68 to 184) | 0.84 (-0.09 to 1.83) | 1.25 (0.82 to 1.77) | 1.22 (0.67 to 1.81) | -0.32 (-0.49 to -0.15) | 1.67 (1.15 to 2.28) | 1.44 (0.83 to 2.12) | -0.74 (-0.83 to -0.64) |
| Thailand | 506 (396 to 632) | 1506 (954 to 2094) | 1.97 (1.01 to 3.43) | 0.89 (0.7 to 1.11) | 2.26 (1.43 to 3.14) | 3.45 (3.32 to 3.58) | 1.03 (0.82 to 1.29) | 1.71 (1.09 to 2.37) | 2.03 (1.87 to 2.19) |
| Timor-Leste | 3 (2 to 5) | 10 (5 to 17) | 2.03 (0.86 to 3.67) | 0.44 (0.24 to 0.65) | 0.74 (0.33 to 1.2) | 2.02 (1.81 to 2.23) | 0.7 (0.4 to 0.99) | 0.93 (0.41 to 1.5) | 1.09 (0.95 to 1.24) |
| Togo | 22 (15 to 31) | 50 (32 to 74) | 1.3 (0.5 to 2.36) | 0.59 (0.41 to 0.85) | 0.59 (0.38 to 0.88) | -0.06 (-0.16 to 0.04) | 0.69 (0.49 to 1) | 0.67 (0.44 to 0.98) | -0.15 (-0.22 to -0.09) |
| Tokelau | 0 (0 to 0) | 0 (0 to 0) | 1.39 (0.53 to 2.62) | 0.48 (0.17 to 0.96) | 1.33 (0.41 to 2.64) | 1.97 (1.46 to 2.5) | 0.51 (0.18 to 1.03) | 1.28 (0.39 to 2.52) | 1.51 (0.97 to 2.06) |
| Tonga | 0 (0 to 1) | 1 (0 to 1) | 0.79 (0.05 to 1.69) | 0.34 (0.14 to 0.6) | 0.56 (0.17 to 1.12) | 1.5 (1.27 to 1.72) | 0.42 (0.18 to 0.74) | 0.63 (0.19 to 1.23) | 1.15 (0.95 to 1.36) |
| Trinidad and Tobago | 10 (9 to 10) | 13 (10 to 16) | 0.32 (0.01 to 0.68) | 0.81 (0.75 to 0.87) | 0.92 (0.71 to 1.17) | 0.53 (0.35 to 0.71) | 0.94 (0.88 to 1) | 0.8 (0.62 to 1) | -0.43 (-0.61 to -0.25) |
| Tunisia | 59 (45 to 77) | 116 (80 to 167) | 0.96 (0.26 to 2.05) | 0.7 (0.54 to 0.92) | 0.98 (0.67 to 1.41) | 1.05 (0.99 to 1.1) | 0.79 (0.61 to 1.02) | 0.95 (0.66 to 1.38) | 0.67 (0.62 to 0.72) |
| Turkmenistan | 48 (33 to 63) | 68 (43 to 104) | 0.41 (-0.13 to 1.24) | 1.3 (0.9 to 1.71) | 1.32 (0.84 to 2.01) | -0.24 (-0.34 to -0.14) | 1.73 (1.24 to 2.29) | 1.44 (0.93 to 2.17) | -0.87 (-0.99 to -0.76) |
| Tuvalu | 0 (0 to 0) | 0 (0 to 0) | 0.89 (0.16 to 1.72) | 0.4 (0.14 to 0.78) | 0.58 (0.16 to 1.15) | 1.06 (0.9 to 1.22) | 0.48 (0.17 to 0.94) | 0.61 (0.17 to 1.21) | 0.7 (0.57 to 0.82) |
| Uganda | 172 (125 to 229) | 511 (348 to 760) | 1.97 (0.8 to 3.82) | 0.99 (0.72 to 1.33) | 1.18 (0.8 to 1.75) | -0.04 (-0.27 to 0.2) | 1.36 (1 to 1.75) | 1.44 (0.99 to 2.17) | -0.36 (-0.6 to -0.13) |
| Ukraine | 553 (486 to 613) | 493 (364 to 641) | -0.11 (-0.36 to 0.2) | 1.05 (0.92 to 1.16) | 1.14 (0.85 to 1.49) | 0.05 (-0.12 to 0.22) | 0.96 (0.83 to 1.09) | 0.97 (0.72 to 1.26) | -0.08 (-0.19 to 0.04) |
| United Arab Emirates | 10 (7 to 13) | 45 (34 to 62) | 3.77 (2.33 to 5.44) | 0.51 (0.36 to 0.68) | 0.47 (0.35 to 0.64) | -0.07 (-0.22 to 0.08) | 0.94 (0.66 to 1.26) | 0.83 (0.63 to 1.09) | 0.85 (0.5 to 1.2) |
| United Kingdom | 548 (534 to 559) | 793 (754 to 824) | 0.45 (0.4 to 0.5) | 0.96 (0.93 to 0.98) | 1.17 (1.11 to 1.21) | 0.83 (0.6 to 1.06) | 0.91 (0.89 to 0.93) | 1.01 (0.97 to 1.05) | 0.48 (0.27 to 0.7) |
| United Republic of Tanzania | 296 (200 to 441) | 662 (370 to 1187) | 1.23 (0.33 to 2.1) | 1.15 (0.77 to 1.71) | 1.13 (0.63 to 2.03) | 0.01 (-0.09 to 0.12) | 1.43 (1.04 to 2.09) | 1.34 (0.78 to 2.34) | -0.24 (-0.3 to -0.18) |
| United States of America | 2133 (2067 to 2188) | 3560 (3372 to 3710) | 0.67 (0.61 to 0.72) | 0.84 (0.81 to 0.86) | 1.07 (1.01 to 1.12) | 0.64 (0.53 to 0.75) | 0.8 (0.78 to 0.82) | 0.92 (0.87 to 0.95) | 0.31 (0.23 to 0.39) |
| United States Virgin Islands | 0 (0 to 0) | 0 (0 to 1) | 0.25 (-0.19 to 0.95) | 0.25 (0.16 to 0.35) | 0.38 (0.2 to 0.62) | 2 (1.84 to 2.17) | 0.28 (0.18 to 0.39) | 0.25 (0.13 to 0.41) | 0.46 (0.24 to 0.67) |
| Uruguay | 10 (9 to 11) | 10 (9 to 11) | 0.01 (-0.15 to 0.19) | 0.32 (0.28 to 0.36) | 0.29 (0.26 to 0.33) | -0.38 (-0.77 to 0) | 0.29 (0.25 to 0.33) | 0.24 (0.21 to 0.27) | -0.79 (-1.18 to -0.4) |
| Uzbekistan | 181 (140 to 229) | 433 (350 to 523) | 1.39 (0.72 to 2.39) | 0.86 (0.67 to 1.09) | 1.26 (1.02 to 1.53) | 1.63 (1.18 to 2.09) | 1.11 (0.83 to 1.43) | 1.38 (1.12 to 1.67) | 1.09 (0.64 to 1.53) |
| Vanuatu | 0 (0 to 1) | 1 (0 to 3) | 2.36 (1.33 to 3.97) | 0.27 (0.08 to 0.58) | 0.45 (0.12 to 0.91) | 1.42 (1.26 to 1.57) | 0.39 (0.12 to 0.84) | 0.56 (0.15 to 1.12) | 1.03 (0.89 to 1.17) |
| Venezuela (Bolivarian Republic of) | 150 (135 to 164) | 357 (269 to 452) | 1.37 (0.74 to 2.16) | 0.8 (0.72 to 0.87) | 1.34 (1.01 to 1.7) | 1.92 (1.67 to 2.17) | 1.03 (0.94 to 1.12) | 1.28 (0.97 to 1.62) | 1.02 (0.78 to 1.27) |
| Viet Nam | 504 (375 to 662) | 1278 (907 to 1699) | 1.53 (0.85 to 2.54) | 0.74 (0.55 to 0.97) | 1.27 (0.9 to 1.69) | 1.84 (1.75 to 1.92) | 0.94 (0.69 to 1.26) | 1.28 (0.92 to 1.68) | 1.08 (1.01 to 1.16) |
| Yemen | 51 (24 to 82) | 157 (80 to 256) | 2.05 (0.92 to 3.77) | 0.38 (0.18 to 0.6) | 0.47 (0.24 to 0.76) | 0.71 (0.6 to 0.82) | 0.56 (0.29 to 0.88) | 0.62 (0.33 to 1) | 0.42 (0.34 to 0.51) |
| Zambia | 86 (60 to 128) | 205 (112 to 364) | 1.39 (0.42 to 2.63) | 1.08 (0.76 to 1.61) | 1.05 (0.58 to 1.87) | -0.16 (-0.27 to -0.05) | 1.41 (1.02 to 2.06) | 1.32 (0.75 to 2.31) | -0.32 (-0.4 to -0.24) |
| Zimbabwe | 64 (48 to 84) | 125 (84 to 191) | 0.96 (0.2 to 2.01) | 0.62 (0.47 to 0.81) | 0.8 (0.54 to 1.22) | 0.84 (0.42 to 1.25) | 0.97 (0.73 to 1.28) | 1.11 (0.77 to 1.7) | 0.52 (0.13 to 0.91) |

CR, crude rate; ASR, age-standardized rate; EAPC, estimated annual percentage change; UI, uncertainty interval; CI, confidence interval.

Table S2. Death of malignant neoplasm of bone and articular cartilage in 1990 and 2021 for both sexes in countries and territories, with EAPC from 1990 to 2021.

| **Location** | **Number** | | | **CR, per 100k** | | | **ASR, per 100k** | | |
| --- | --- | --- | --- | --- | --- | --- | --- | --- | --- |
|  | **Number in 1990 (95% UI)** | **Number in 2021 (95% UI)** | **Number change rate (95% UI)** | **CR in 1990 (95% UI)** | **CR in 2021 (95% UI)** | **EAPC of CR, % per year (95% CI)** | **ASR in 1990 (95% UI)** | **ASR in 2021 (95% UI)** | **EAPC of ASR, % per year (95% CI)** |
| Afghanistan | 85 (50 to 133) | 205 (116 to 316) | 1.41 (0.64 to 2.46) | 0.86 (0.5 to 1.34) | 0.66 (0.37 to 1.01) | -0.62 (-0.96 to -0.27) | 1.05 (0.64 to 1.59) | 1.12 (0.69 to 1.65) | 0.35 (0.21 to 0.48) |
| Albania | 39 (28 to 53) | 41 (21 to 69) | 0.04 (-0.45 to 0.55) | 1.18 (0.86 to 1.6) | 1.52 (0.77 to 2.57) | 0.85 (0.71 to 1) | 1.58 (1.13 to 2.16) | 1.03 (0.55 to 1.73) | -1.4 (-1.57 to -1.23) |
| Algeria | 169 (118 to 216) | 300 (213 to 433) | 0.78 (0.16 to 1.78) | 0.67 (0.47 to 0.85) | 0.68 (0.48 to 0.98) | 0 (-0.08 to 0.08) | 0.94 (0.69 to 1.18) | 0.83 (0.59 to 1.2) | -0.36 (-0.45 to -0.27) |
| American Samoa | 0 (0 to 0) | 1 (0 to 1) | 1.04 (0.27 to 2.35) | 0.51 (0.36 to 0.74) | 1.02 (0.74 to 1.35) | 2.76 (2.51 to 3.01) | 0.77 (0.55 to 1.07) | 1.04 (0.76 to 1.37) | 1.46 (1.17 to 1.76) |
| Andorra | 0 (0 to 0) | 0 (0 to 0) | 0.38 (-0.25 to 1.48) | 0.09 (0.03 to 0.17) | 0.08 (0.03 to 0.15) | -0.38 (-0.57 to -0.18) | 0.09 (0.03 to 0.17) | 0.05 (0.02 to 0.1) | -1.49 (-1.65 to -1.33) |
| Angola | 45 (29 to 63) | 125 (78 to 194) | 1.81 (0.69 to 3.58) | 0.43 (0.29 to 0.61) | 0.38 (0.24 to 0.59) | -0.4 (-0.54 to -0.26) | 0.76 (0.5 to 1.07) | 0.68 (0.44 to 1) | -0.4 (-0.5 to -0.3) |
| Antigua and Barbuda | 0 (0 to 0) | 0 (0 to 1) | 0.66 (0.47 to 0.92) | 0.48 (0.42 to 0.53) | 0.54 (0.51 to 0.57) | 0.7 (0.32 to 1.09) | 0.5 (0.45 to 0.56) | 0.49 (0.46 to 0.51) | 0.18 (-0.06 to 0.43) |
| Argentina | 462 (402 to 515) | 421 (386 to 462) | -0.09 (-0.2 to 0.04) | 1.4 (1.21 to 1.56) | 0.93 (0.85 to 1.01) | -1.35 (-1.58 to -1.12) | 1.43 (1.24 to 1.59) | 0.8 (0.73 to 0.87) | -1.85 (-2.08 to -1.62) |
| Armenia | 25 (18 to 33) | 31 (22 to 42) | 0.22 (-0.25 to 0.88) | 0.74 (0.53 to 0.98) | 1.02 (0.74 to 1.4) | 1.42 (1.18 to 1.66) | 0.86 (0.62 to 1.14) | 0.76 (0.55 to 1.03) | -0.14 (-0.37 to 0.09) |
| Australia | 98 (92 to 105) | 110 (98 to 121) | 0.13 (0 to 0.26) | 0.58 (0.55 to 0.62) | 0.43 (0.38 to 0.47) | -1 (-1.26 to -0.75) | 0.53 (0.5 to 0.57) | 0.32 (0.28 to 0.35) | -1.74 (-2.04 to -1.45) |
| Austria | 48 (44 to 51) | 53 (47 to 60) | 0.12 (-0.02 to 0.27) | 0.61 (0.57 to 0.66) | 0.59 (0.53 to 0.67) | -0.47 (-0.8 to -0.14) | 0.47 (0.43 to 0.5) | 0.35 (0.32 to 0.39) | -1.4 (-1.71 to -1.08) |
| Azerbaijan | 37 (22 to 63) | 55 (34 to 91) | 0.49 (-0.16 to 1.54) | 0.51 (0.3 to 0.86) | 0.52 (0.33 to 0.86) | 0.09 (0.02 to 0.16) | 0.63 (0.38 to 1.04) | 0.52 (0.33 to 0.85) | -0.58 (-0.65 to -0.51) |
| Bahamas | 1 (1 to 1) | 2 (2 to 3) | 0.72 (0.37 to 1.19) | 0.51 (0.46 to 0.56) | 0.58 (0.47 to 0.72) | 0.47 (0.16 to 0.78) | 0.67 (0.61 to 0.72) | 0.56 (0.45 to 0.69) | -0.53 (-0.8 to -0.25) |
| Bahrain | 3 (2 to 4) | 9 (7 to 12) | 2.47 (1.52 to 3.9) | 0.53 (0.41 to 0.69) | 0.6 (0.45 to 0.81) | 0.28 (0.02 to 0.54) | 1.06 (0.8 to 1.41) | 0.95 (0.69 to 1.28) | -0.38 (-0.52 to -0.23) |
| Bangladesh | 653 (459 to 878) | 1207 (829 to 1676) | 0.85 (0.19 to 1.69) | 0.6 (0.42 to 0.8) | 0.73 (0.5 to 1.02) | 0.76 (0.63 to 0.88) | 0.82 (0.59 to 1.09) | 0.78 (0.54 to 1.08) | -0.19 (-0.24 to -0.14) |
| Barbados | 2 (2 to 2) | 3 (2 to 3) | 0.32 (0.03 to 0.6) | 0.86 (0.78 to 0.93) | 0.96 (0.77 to 1.14) | 0.72 (0.54 to 0.9) | 0.75 (0.69 to 0.81) | 0.63 (0.5 to 0.75) | -0.17 (-0.35 to 0.01) |
| Belarus | 119 (107 to 133) | 73 (56 to 93) | -0.38 (-0.54 to -0.22) | 1.14 (1.02 to 1.27) | 0.79 (0.6 to 0.99) | -1.45 (-1.56 to -1.35) | 0.99 (0.89 to 1.1) | 0.54 (0.4 to 0.69) | -2.19 (-2.29 to -2.09) |
| Belgium | 106 (99 to 114) | 93 (81 to 105) | -0.13 (-0.22 to -0.02) | 1.06 (0.99 to 1.15) | 0.81 (0.71 to 0.92) | -1.04 (-1.41 to -0.67) | 0.81 (0.75 to 0.87) | 0.48 (0.43 to 0.54) | -1.92 (-2.29 to -1.55) |
| Belize | 1 (1 to 1) | 2 (2 to 2) | 1.36 (0.92 to 2.02) | 0.49 (0.39 to 0.58) | 0.51 (0.45 to 0.57) | 0.3 (0.07 to 0.54) | 0.7 (0.54 to 0.82) | 0.63 (0.55 to 0.7) | -0.16 (-0.46 to 0.15) |
| Benin | 21 (16 to 30) | 54 (38 to 76) | 1.54 (0.79 to 2.7) | 0.44 (0.33 to 0.61) | 0.4 (0.28 to 0.56) | -0.4 (-0.5 to -0.31) | 0.62 (0.45 to 0.85) | 0.57 (0.4 to 0.78) | -0.41 (-0.5 to -0.32) |
| Bermuda | 0 (0 to 0) | 0 (0 to 1) | 0.25 (-0.02 to 0.65) | 0.57 (0.47 to 0.65) | 0.67 (0.55 to 0.82) | 0.6 (0.23 to 0.97) | 0.55 (0.46 to 0.63) | 0.35 (0.29 to 0.42) | -1.47 (-1.72 to -1.21) |
| Bhutan | 3 (2 to 4) | 6 (4 to 9) | 0.89 (0.16 to 2.04) | 0.5 (0.33 to 0.71) | 0.79 (0.55 to 1.13) | 1.37 (1.25 to 1.49) | 0.72 (0.5 to 1.01) | 0.86 (0.6 to 1.23) | 0.51 (0.45 to 0.58) |
| Bolivia (Plurinational State of) | 55 (41 to 75) | 123 (78 to 181) | 1.22 (0.33 to 2.57) | 0.87 (0.64 to 1.17) | 1.04 (0.66 to 1.53) | 0.59 (0.55 to 0.63) | 1.22 (0.92 to 1.61) | 1.23 (0.8 to 1.8) | -0.05 (-0.09 to -0.01) |
| Bosnia and Herzegovina | 31 (17 to 49) | 28 (18 to 43) | -0.07 (-0.48 to 0.45) | 0.68 (0.37 to 1.1) | 0.86 (0.56 to 1.31) | 0.53 (0.38 to 0.68) | 0.72 (0.4 to 1.17) | 0.52 (0.34 to 0.77) | -1.19 (-1.29 to -1.1) |
| Botswana | 7 (5 to 10) | 17 (12 to 22) | 1.32 (0.45 to 2.51) | 0.55 (0.37 to 0.76) | 0.7 (0.49 to 0.92) | 0.87 (0.72 to 1.01) | 0.9 (0.62 to 1.24) | 0.93 (0.67 to 1.22) | 0.2 (0.06 to 0.34) |
| Brazil | 1322 (1251 to 1376) | 2387 (2248 to 2507) | 0.81 (0.71 to 0.91) | 0.89 (0.84 to 0.93) | 1.08 (1.02 to 1.14) | 0.87 (0.74 to 0.99) | 1.25 (1.19 to 1.31) | 0.99 (0.93 to 1.04) | -0.51 (-0.64 to -0.38) |
| Brunei Darussalam | 1 (0 to 1) | 1 (1 to 1) | 0.75 (0.19 to 1.57) | 0.23 (0.14 to 0.34) | 0.23 (0.15 to 0.33) | 0.49 (0.26 to 0.72) | 0.29 (0.19 to 0.42) | 0.25 (0.16 to 0.35) | 0 (-0.19 to 0.19) |
| Bulgaria | 103 (85 to 122) | 89 (70 to 112) | -0.14 (-0.36 to 0.17) | 1.19 (0.98 to 1.4) | 1.31 (1.03 to 1.65) | 0.67 (0.31 to 1.04) | 0.96 (0.79 to 1.13) | 0.75 (0.58 to 0.93) | -0.48 (-0.84 to -0.12) |
| Burkina Faso | 40 (28 to 58) | 84 (58 to 115) | 1.1 (0.43 to 2.07) | 0.42 (0.29 to 0.61) | 0.37 (0.25 to 0.5) | -0.4 (-0.6 to -0.2) | 0.6 (0.4 to 0.9) | 0.52 (0.36 to 0.73) | -0.47 (-0.63 to -0.31) |
| Burundi | 45 (31 to 67) | 88 (50 to 149) | 0.94 (0.25 to 1.84) | 0.82 (0.57 to 1.21) | 0.67 (0.38 to 1.13) | -0.79 (-0.85 to -0.73) | 1.2 (0.84 to 1.75) | 1.01 (0.59 to 1.67) | -0.7 (-0.76 to -0.63) |
| Cabo Verde | 1 (1 to 2) | 2 (1 to 3) | 0.86 (0.25 to 1.63) | 0.3 (0.2 to 0.45) | 0.35 (0.23 to 0.48) | 0.5 (0.45 to 0.55) | 0.35 (0.23 to 0.51) | 0.39 (0.25 to 0.53) | 0.27 (0.21 to 0.33) |
| Cambodia | 51 (29 to 70) | 148 (62 to 237) | 1.9 (0.68 to 3.4) | 0.5 (0.28 to 0.69) | 0.87 (0.36 to 1.39) | 1.98 (1.92 to 2.03) | 0.84 (0.49 to 1.14) | 1.09 (0.46 to 1.7) | 0.92 (0.87 to 0.96) |
| Cameroon | 57 (40 to 77) | 152 (102 to 225) | 1.67 (0.8 to 2.81) | 0.54 (0.39 to 0.73) | 0.48 (0.32 to 0.71) | -0.42 (-0.49 to -0.36) | 0.78 (0.55 to 1.04) | 0.67 (0.45 to 0.98) | -0.5 (-0.56 to -0.44) |
| Canada | 149 (140 to 157) | 199 (178 to 220) | 0.34 (0.2 to 0.48) | 0.55 (0.52 to 0.58) | 0.53 (0.47 to 0.59) | -0.03 (-0.25 to 0.2) | 0.5 (0.47 to 0.52) | 0.38 (0.34 to 0.42) | -0.92 (-1.18 to -0.67) |
| Central African Republic | 13 (9 to 18) | 22 (13 to 33) | 0.7 (0.13 to 1.37) | 0.48 (0.33 to 0.66) | 0.4 (0.24 to 0.6) | -0.63 (-0.74 to -0.52) | 0.81 (0.57 to 1.14) | 0.67 (0.4 to 0.96) | -0.7 (-0.76 to -0.65) |
| Chad | 23 (16 to 34) | 68 (44 to 98) | 1.97 (0.92 to 3.31) | 0.38 (0.26 to 0.57) | 0.38 (0.25 to 0.55) | 0.13 (0.07 to 0.19) | 0.52 (0.36 to 0.79) | 0.57 (0.36 to 0.82) | 0.35 (0.3 to 0.4) |
| Chile | 165 (151 to 180) | 166 (152 to 180) | 0.01 (-0.09 to 0.12) | 1.24 (1.13 to 1.35) | 0.88 (0.81 to 0.96) | -1.04 (-1.29 to -0.79) | 1.47 (1.34 to 1.61) | 0.71 (0.65 to 0.77) | -2.37 (-2.6 to -2.14) |
| China | 5292 (3393 to 9393) | 18085 (11288 to 24126) | 2.42 (0.51 to 5.56) | 0.45 (0.29 to 0.8) | 1.27 (0.79 to 1.7) | 4.19 (3.56 to 4.82) | 0.58 (0.38 to 1.03) | 0.93 (0.58 to 1.23) | 2.21 (1.53 to 2.89) |
| Colombia | 212 (199 to 225) | 417 (351 to 492) | 0.97 (0.65 to 1.33) | 0.65 (0.61 to 0.69) | 0.85 (0.71 to 1) | 0.81 (0.64 to 0.98) | 0.92 (0.86 to 0.98) | 0.78 (0.66 to 0.92) | -0.74 (-0.89 to -0.58) |
| Comoros | 4 (3 to 6) | 8 (5 to 14) | 0.95 (0.15 to 2.54) | 0.87 (0.6 to 1.23) | 1.05 (0.61 to 1.92) | 0.09 (-0.34 to 0.52) | 1.26 (0.88 to 1.8) | 1.26 (0.73 to 2.28) | -0.41 (-0.69 to -0.12) |
| Congo | 13 (9 to 17) | 29 (20 to 46) | 1.3 (0.54 to 2.32) | 0.53 (0.38 to 0.72) | 0.55 (0.37 to 0.85) | -0.12 (-0.26 to 0.02) | 0.89 (0.64 to 1.16) | 0.81 (0.57 to 1.24) | -0.44 (-0.55 to -0.33) |
| Cook Islands | 0 (0 to 0) | 0 (0 to 0) | 0.33 (-0.11 to 0.91) | 0.19 (0.11 to 0.29) | 0.27 (0.15 to 0.42) | 1.05 (0.89 to 1.21) | 0.24 (0.14 to 0.35) | 0.21 (0.11 to 0.33) | -0.53 (-0.67 to -0.4) |
| Costa Rica | 14 (13 to 16) | 37 (32 to 42) | 1.54 (1.14 to 1.95) | 0.47 (0.43 to 0.51) | 0.77 (0.67 to 0.88) | 1.74 (1.36 to 2.11) | 0.63 (0.57 to 0.68) | 0.69 (0.6 to 0.79) | 0.39 (0.1 to 0.68) |
| Côte d'Ivoire | 43 (29 to 61) | 104 (69 to 142) | 1.42 (0.73 to 2.35) | 0.35 (0.24 to 0.5) | 0.37 (0.25 to 0.51) | 0.23 (0.12 to 0.33) | 0.53 (0.36 to 0.72) | 0.51 (0.35 to 0.7) | -0.08 (-0.16 to 0) |
| Croatia | 60 (52 to 68) | 59 (50 to 69) | -0.01 (-0.19 to 0.17) | 1.23 (1.08 to 1.4) | 1.41 (1.19 to 1.64) | 0.01 (-0.43 to 0.45) | 1.07 (0.94 to 1.21) | 0.74 (0.62 to 0.87) | -1.58 (-1.97 to -1.18) |
| Cuba | 126 (116 to 137) | 227 (196 to 260) | 0.79 (0.52 to 1.09) | 1.17 (1.07 to 1.27) | 2.01 (1.74 to 2.31) | 1.59 (1.38 to 1.81) | 1.18 (1.08 to 1.28) | 1.27 (1.1 to 1.45) | -0.03 (-0.23 to 0.17) |
| Cyprus | 6 (4 to 8) | 9 (6 to 12) | 0.48 (0.03 to 1.3) | 0.76 (0.51 to 1.06) | 0.64 (0.46 to 0.85) | -0.44 (-0.58 to -0.29) | 0.79 (0.53 to 1.12) | 0.48 (0.35 to 0.64) | -1.45 (-1.6 to -1.29) |
| Czechia | 97 (88 to 107) | 68 (57 to 82) | -0.3 (-0.43 to -0.12) | 0.94 (0.86 to 1.04) | 0.64 (0.54 to 0.77) | -1.02 (-1.28 to -0.75) | 0.76 (0.69 to 0.84) | 0.37 (0.3 to 0.45) | -2.09 (-2.37 to -1.81) |
| Democratic People's Republic of Korea | 152 (85 to 224) | 330 (157 to 532) | 1.18 (0.38 to 2.42) | 0.74 (0.41 to 1.09) | 1.25 (0.59 to 2.02) | 1.9 (1.77 to 2.04) | 0.89 (0.51 to 1.3) | 1.05 (0.5 to 1.68) | 0.69 (0.54 to 0.84) |
| Democratic Republic of the Congo | 174 (121 to 237) | 345 (206 to 522) | 0.98 (0.2 to 1.87) | 0.46 (0.32 to 0.62) | 0.38 (0.23 to 0.58) | -0.66 (-0.84 to -0.49) | 0.8 (0.55 to 1.07) | 0.64 (0.39 to 0.95) | -0.78 (-0.92 to -0.64) |
| Denmark | 30 (28 to 33) | 29 (25 to 32) | -0.06 (-0.16 to 0.06) | 0.59 (0.55 to 0.63) | 0.49 (0.43 to 0.54) | -1.36 (-1.72 to -1) | 0.43 (0.4 to 0.46) | 0.28 (0.24 to 0.31) | -2.18 (-2.55 to -1.8) |
| Djibouti | 3 (2 to 5) | 11 (6 to 20) | 2.88 (1.23 to 5.18) | 0.69 (0.42 to 1.09) | 0.88 (0.49 to 1.6) | 0.7 (0.47 to 0.93) | 1.09 (0.68 to 1.67) | 1.22 (0.69 to 2.16) | 0.34 (0.18 to 0.49) |
| Dominica | 1 (0 to 1) | 1 (1 to 1) | 0.5 (0.05 to 1.17) | 0.74 (0.54 to 1.01) | 1.2 (0.9 to 1.54) | 1.75 (1.6 to 1.89) | 0.84 (0.6 to 1.15) | 1.04 (0.79 to 1.33) | 0.86 (0.69 to 1.03) |
| Dominican Republic | 52 (35 to 76) | 120 (83 to 178) | 1.3 (0.66 to 2.51) | 0.73 (0.49 to 1.07) | 1.09 (0.75 to 1.61) | 1.66 (1.52 to 1.81) | 1.05 (0.67 to 1.57) | 1.17 (0.8 to 1.72) | 0.67 (0.53 to 0.81) |
| Ecuador | 113 (97 to 132) | 156 (125 to 191) | 0.38 (0.05 to 0.82) | 1.14 (0.98 to 1.33) | 0.86 (0.69 to 1.06) | -0.68 (-0.84 to -0.53) | 1.61 (1.4 to 1.86) | 0.94 (0.76 to 1.15) | -1.58 (-1.73 to -1.43) |
| Egypt | 470 (352 to 614) | 995 (710 to 1441) | 1.12 (0.45 to 1.94) | 0.85 (0.64 to 1.11) | 0.94 (0.67 to 1.36) | 0.28 (0.12 to 0.44) | 1.4 (1.01 to 1.84) | 1.46 (1.04 to 2.13) | 0.22 (0.06 to 0.39) |
| El Salvador | 19 (15 to 22) | 38 (24 to 53) | 1.03 (0.37 to 1.86) | 0.36 (0.28 to 0.42) | 0.6 (0.36 to 0.82) | 1.87 (1.75 to 1.99) | 0.48 (0.37 to 0.57) | 0.6 (0.37 to 0.83) | 0.78 (0.7 to 0.85) |
| Equatorial Guinea | 2 (1 to 3) | 7 (4 to 11) | 2.29 (0.96 to 4.91) | 0.48 (0.31 to 0.68) | 0.44 (0.27 to 0.7) | -0.25 (-0.38 to -0.13) | 0.78 (0.51 to 1.13) | 0.75 (0.46 to 1.17) | -0.04 (-0.12 to 0.03) |
| Eritrea | 26 (17 to 38) | 55 (30 to 94) | 1.13 (0.32 to 2) | 0.76 (0.51 to 1.12) | 0.83 (0.46 to 1.42) | 0.13 (0.07 to 0.2) | 1.2 (0.84 to 1.73) | 1.19 (0.68 to 2) | -0.16 (-0.22 to -0.1) |
| Estonia | 13 (11 to 14) | 6 (5 to 7) | -0.52 (-0.61 to -0.39) | 0.81 (0.7 to 0.91) | 0.47 (0.38 to 0.55) | -3.03 (-3.61 to -2.46) | 0.69 (0.6 to 0.77) | 0.26 (0.21 to 0.3) | -4.39 (-4.95 to -3.83) |
| Eswatini | 5 (3 to 6) | 10 (6 to 14) | 0.98 (0.33 to 1.92) | 0.6 (0.43 to 0.79) | 0.83 (0.5 to 1.25) | 1.11 (0.75 to 1.47) | 1.12 (0.82 to 1.47) | 1.26 (0.78 to 1.86) | 0.43 (0.06 to 0.81) |
| Ethiopia | 610 (457 to 825) | 991 (712 to 1433) | 0.63 (0.13 to 1.49) | 1.21 (0.9 to 1.63) | 0.91 (0.65 to 1.32) | -1.33 (-1.52 to -1.14) | 1.86 (1.42 to 2.44) | 1.33 (0.98 to 1.88) | -1.48 (-1.64 to -1.33) |
| Fiji | 4 (2 to 6) | 7 (5 to 10) | 0.81 (0.25 to 1.9) | 0.52 (0.33 to 0.79) | 0.77 (0.53 to 1.1) | 1.32 (1.01 to 1.63) | 0.71 (0.45 to 1.08) | 0.86 (0.6 to 1.22) | 0.58 (0.28 to 0.88) |
| Finland | 30 (27 to 32) | 16 (14 to 18) | -0.45 (-0.52 to -0.37) | 0.59 (0.55 to 0.64) | 0.29 (0.25 to 0.33) | -2.38 (-2.61 to -2.14) | 0.47 (0.43 to 0.5) | 0.17 (0.15 to 0.19) | -3.44 (-3.66 to -3.22) |
| France | 739 (675 to 780) | 653 (575 to 733) | -0.12 (-0.2 to -0.03) | 1.28 (1.17 to 1.35) | 0.98 (0.87 to 1.1) | -0.86 (-1.06 to -0.66) | 1 (0.92 to 1.05) | 0.58 (0.51 to 0.64) | -1.79 (-1.94 to -1.64) |
| Gabon | 6 (4 to 7) | 10 (7 to 16) | 0.78 (0.13 to 1.78) | 0.58 (0.42 to 0.75) | 0.56 (0.38 to 0.86) | -0.31 (-0.4 to -0.22) | 0.83 (0.6 to 1.11) | 0.79 (0.54 to 1.19) | -0.37 (-0.52 to -0.23) |
| Gambia | 4 (3 to 6) | 13 (9 to 20) | 2.13 (0.99 to 3.68) | 0.43 (0.31 to 0.57) | 0.55 (0.36 to 0.84) | 0.54 (0.28 to 0.79) | 0.62 (0.45 to 0.82) | 0.73 (0.49 to 1.08) | 0.29 (0.08 to 0.5) |
| Georgia | 55 (40 to 73) | 74 (57 to 96) | 0.34 (-0.1 to 1.04) | 1 (0.72 to 1.32) | 2.06 (1.58 to 2.65) | 2.58 (2 to 3.16) | 0.91 (0.65 to 1.2) | 1.42 (1.13 to 1.8) | 1.66 (1.11 to 2.21) |
| Germany | 422 (383 to 466) | 446 (399 to 492) | 0.06 (-0.06 to 0.18) | 0.53 (0.48 to 0.58) | 0.52 (0.47 to 0.58) | -0.2 (-0.36 to -0.04) | 0.39 (0.36 to 0.43) | 0.31 (0.28 to 0.33) | -1.18 (-1.43 to -0.94) |
| Ghana | 62 (42 to 93) | 155 (103 to 222) | 1.51 (0.55 to 3.06) | 0.41 (0.28 to 0.62) | 0.45 (0.3 to 0.65) | 0.28 (0.23 to 0.34) | 0.56 (0.37 to 0.85) | 0.6 (0.4 to 0.85) | 0.2 (0.15 to 0.24) |
| Greece | 262 (246 to 275) | 178 (160 to 191) | -0.32 (-0.37 to -0.27) | 2.52 (2.37 to 2.64) | 1.75 (1.58 to 1.88) | -1.83 (-2.11 to -1.55) | 1.87 (1.77 to 1.97) | 0.93 (0.87 to 1) | -2.84 (-3.11 to -2.57) |
| Greenland | 0 (0 to 0) | 0 (0 to 0) | -0.4 (-0.68 to 0.22) | 0.45 (0.22 to 0.71) | 0.27 (0.19 to 0.38) | -1.3 (-1.73 to -0.88) | 0.55 (0.28 to 0.85) | 0.25 (0.17 to 0.35) | -2.38 (-2.77 to -1.98) |
| Grenada | 1 (1 to 1) | 1 (1 to 1) | 0.01 (-0.18 to 0.28) | 0.87 (0.73 to 1.01) | 0.75 (0.65 to 0.85) | 0.23 (-0.08 to 0.54) | 0.97 (0.82 to 1.12) | 0.69 (0.6 to 0.78) | -0.54 (-0.95 to -0.12) |
| Guam | 0 (0 to 0) | 0 (0 to 1) | 0.54 (-0.12 to 0.99) | 0.18 (0.12 to 0.25) | 0.24 (0.15 to 0.34) | 1.53 (1.28 to 1.78) | 0.26 (0.17 to 0.36) | 0.21 (0.13 to 0.29) | -0.16 (-0.41 to 0.1) |
| Guatemala | 51 (43 to 57) | 100 (86 to 116) | 0.97 (0.62 to 1.43) | 0.61 (0.52 to 0.68) | 0.63 (0.54 to 0.74) | 0.65 (0.41 to 0.88) | 1.06 (0.92 to 1.15) | 0.79 (0.68 to 0.92) | -0.65 (-0.83 to -0.47) |
| Guinea | 35 (26 to 46) | 73 (51 to 103) | 1.07 (0.45 to 2.02) | 0.58 (0.43 to 0.76) | 0.54 (0.38 to 0.77) | -0.27 (-0.31 to -0.23) | 0.72 (0.54 to 0.93) | 0.72 (0.51 to 1.01) | -0.05 (-0.09 to 0) |
| Guinea-Bissau | 5 (3 to 8) | 9 (6 to 13) | 0.73 (0.2 to 1.54) | 0.53 (0.35 to 0.76) | 0.45 (0.3 to 0.64) | -0.56 (-0.65 to -0.48) | 0.74 (0.49 to 1.09) | 0.64 (0.44 to 0.87) | -0.49 (-0.54 to -0.43) |
| Guyana | 6 (5 to 6) | 7 (5 to 9) | 0.19 (-0.13 to 0.56) | 0.72 (0.61 to 0.83) | 0.88 (0.68 to 1.11) | 1.27 (1.02 to 1.53) | 1.06 (0.93 to 1.2) | 0.96 (0.75 to 1.22) | 0.09 (-0.1 to 0.28) |
| Haiti | 38 (23 to 55) | 73 (42 to 115) | 0.93 (0.27 to 2.09) | 0.59 (0.36 to 0.86) | 0.57 (0.33 to 0.89) | 0.08 (0.01 to 0.15) | 0.88 (0.56 to 1.29) | 0.81 (0.46 to 1.29) | -0.1 (-0.16 to -0.05) |
| Honduras | 14 (9 to 19) | 44 (24 to 68) | 2.24 (1.13 to 4.14) | 0.29 (0.19 to 0.4) | 0.43 (0.24 to 0.68) | 1.3 (1.2 to 1.41) | 0.43 (0.29 to 0.61) | 0.6 (0.33 to 0.93) | 1.04 (0.89 to 1.19) |
| Hungary | 131 (120 to 143) | 58 (48 to 72) | -0.55 (-0.64 to -0.45) | 1.26 (1.16 to 1.37) | 0.61 (0.5 to 0.75) | -2.62 (-3 to -2.23) | 0.98 (0.9 to 1.07) | 0.36 (0.29 to 0.43) | -3.51 (-3.92 to -3.11) |
| Iceland | 2 (1 to 2) | 2 (2 to 3) | 0.57 (0.35 to 0.82) | 0.61 (0.57 to 0.67) | 0.7 (0.6 to 0.79) | -0.46 (-0.97 to 0.05) | 0.56 (0.52 to 0.61) | 0.49 (0.42 to 0.55) | -1.39 (-1.89 to -0.88) |
| India | 4882 (3551 to 5773) | 9849 (8042 to 12420) | 1.02 (0.63 to 1.72) | 0.57 (0.42 to 0.68) | 0.7 (0.57 to 0.88) | 0.51 (0.42 to 0.61) | 0.75 (0.56 to 0.89) | 0.75 (0.61 to 0.94) | -0.13 (-0.21 to -0.04) |
| Indonesia | 859 (505 to 1094) | 2766 (1237 to 4340) | 2.22 (1.02 to 3.51) | 0.46 (0.27 to 0.59) | 0.99 (0.44 to 1.56) | 2.51 (2.39 to 2.62) | 0.67 (0.41 to 0.85) | 1.1 (0.5 to 1.69) | 1.73 (1.57 to 1.89) |
| Iran (Islamic Republic of) | 238 (173 to 284) | 404 (314 to 487) | 0.7 (0.38 to 1.25) | 0.42 (0.3 to 0.5) | 0.47 (0.37 to 0.57) | 0.43 (0.27 to 0.59) | 0.6 (0.47 to 0.73) | 0.53 (0.41 to 0.64) | -0.3 (-0.38 to -0.23) |
| Iraq | 147 (108 to 197) | 351 (258 to 485) | 1.39 (0.69 to 2.55) | 0.8 (0.58 to 1.07) | 0.85 (0.63 to 1.18) | 0.31 (0.25 to 0.37) | 1.22 (0.91 to 1.63) | 1.18 (0.88 to 1.64) | 0 (-0.07 to 0.06) |
| Ireland | 30 (28 to 32) | 25 (21 to 28) | -0.18 (-0.29 to -0.05) | 0.83 (0.76 to 0.9) | 0.5 (0.43 to 0.56) | -1.62 (-2.02 to -1.21) | 0.75 (0.7 to 0.82) | 0.35 (0.31 to 0.4) | -2.21 (-2.52 to -1.9) |
| Israel | 30 (27 to 33) | 38 (33 to 42) | 0.26 (0.1 to 0.43) | 0.6 (0.53 to 0.67) | 0.39 (0.34 to 0.44) | -1.59 (-1.77 to -1.42) | 0.61 (0.54 to 0.68) | 0.33 (0.29 to 0.37) | -2.17 (-2.33 to -2) |
| Italy | 781 (734 to 812) | 516 (459 to 561) | -0.34 (-0.39 to -0.29) | 1.37 (1.29 to 1.43) | 0.86 (0.77 to 0.94) | -1.14 (-1.6 to -0.68) | 1.01 (0.95 to 1.04) | 0.47 (0.43 to 0.51) | -2.02 (-2.46 to -1.58) |
| Jamaica | 22 (17 to 25) | 30 (22 to 38) | 0.37 (-0.02 to 0.82) | 0.91 (0.73 to 1.05) | 1.06 (0.8 to 1.37) | 0.58 (0.24 to 0.92) | 1.07 (0.86 to 1.24) | 0.97 (0.73 to 1.26) | -0.25 (-0.56 to 0.06) |
| Japan | 488 (471 to 501) | 457 (408 to 486) | -0.06 (-0.14 to -0.01) | 0.39 (0.37 to 0.4) | 0.36 (0.32 to 0.38) | -0.11 (-0.38 to 0.15) | 0.34 (0.33 to 0.35) | 0.21 (0.2 to 0.22) | -1.46 (-1.67 to -1.24) |
| Jordan | 22 (16 to 29) | 82 (57 to 109) | 2.76 (1.54 to 4.4) | 0.58 (0.44 to 0.77) | 0.66 (0.46 to 0.88) | 0.29 (0.18 to 0.4) | 0.92 (0.7 to 1.2) | 0.89 (0.62 to 1.18) | -0.13 (-0.25 to -0.02) |
| Kazakhstan | 66 (50 to 84) | 52 (39 to 70) | -0.21 (-0.49 to 0.17) | 0.4 (0.31 to 0.51) | 0.28 (0.21 to 0.37) | -1.7 (-2.17 to -1.22) | 0.47 (0.36 to 0.59) | 0.28 (0.21 to 0.37) | -2.09 (-2.52 to -1.66) |
| Kenya | 178 (135 to 226) | 484 (365 to 694) | 1.72 (1.03 to 2.8) | 0.77 (0.58 to 0.98) | 0.97 (0.73 to 1.39) | 0.87 (0.79 to 0.95) | 1.2 (0.9 to 1.53) | 1.34 (1.02 to 1.91) | 0.5 (0.43 to 0.56) |
| Kiribati | 0 (0 to 0) | 1 (0 to 1) | 1.17 (0.45 to 2.46) | 0.33 (0.22 to 0.45) | 0.44 (0.23 to 0.7) | 1.1 (0.84 to 1.35) | 0.44 (0.3 to 0.6) | 0.55 (0.3 to 0.86) | 0.8 (0.57 to 1.04) |
| Kuwait | 6 (6 to 7) | 17 (14 to 20) | 1.73 (1.24 to 2.33) | 0.36 (0.32 to 0.4) | 0.36 (0.3 to 0.44) | 0.19 (-1.15 to 1.55) | 0.61 (0.55 to 0.68) | 0.52 (0.43 to 0.62) | -0.02 (-1.38 to 1.35) |
| Kyrgyzstan | 42 (32 to 54) | 90 (67 to 121) | 1.17 (0.39 to 2.29) | 0.94 (0.72 to 1.22) | 1.32 (0.97 to 1.76) | 1.18 (0.74 to 1.63) | 1.18 (0.91 to 1.53) | 1.65 (1.23 to 2.18) | 1.18 (0.72 to 1.65) |
| Lao People's Democratic Republic | 23 (12 to 33) | 60 (26 to 102) | 1.61 (0.53 to 3.26) | 0.55 (0.29 to 0.79) | 0.81 (0.35 to 1.38) | 1.37 (1.32 to 1.43) | 0.86 (0.48 to 1.23) | 1.1 (0.48 to 1.85) | 0.85 (0.73 to 0.96) |
| Latvia | 28 (26 to 32) | 17 (14 to 21) | -0.39 (-0.52 to -0.26) | 1.07 (0.96 to 1.19) | 0.92 (0.77 to 1.11) | -1.32 (-1.79 to -0.86) | 0.9 (0.8 to 1) | 0.5 (0.42 to 0.61) | -2.73 (-3.2 to -2.26) |
| Lebanon | 20 (15 to 26) | 43 (32 to 61) | 1.17 (0.44 to 2.09) | 0.67 (0.49 to 0.88) | 0.78 (0.57 to 1.1) | 0.6 (0.35 to 0.85) | 0.83 (0.62 to 1.1) | 0.71 (0.52 to 1) | -0.44 (-0.6 to -0.27) |
| Lesotho | 8 (5 to 10) | 15 (11 to 21) | 1.01 (0.29 to 2.13) | 0.5 (0.35 to 0.65) | 0.82 (0.57 to 1.14) | 1.99 (1.69 to 2.3) | 0.75 (0.53 to 1) | 1.15 (0.8 to 1.6) | 1.88 (1.58 to 2.18) |
| Liberia | 13 (9 to 17) | 24 (17 to 36) | 0.9 (0.32 to 1.75) | 0.52 (0.36 to 0.68) | 0.44 (0.31 to 0.65) | -0.53 (-0.85 to -0.21) | 0.7 (0.48 to 0.93) | 0.61 (0.42 to 0.89) | -0.23 (-0.47 to 0.01) |
| Libya | 18 (13 to 24) | 41 (29 to 59) | 1.33 (0.45 to 2.52) | 0.42 (0.31 to 0.56) | 0.6 (0.42 to 0.86) | 1.31 (1.24 to 1.39) | 0.63 (0.45 to 0.85) | 0.7 (0.49 to 1) | 0.65 (0.54 to 0.77) |
| Lithuania | 31 (28 to 34) | 16 (13 to 19) | -0.49 (-0.58 to -0.39) | 0.84 (0.76 to 0.93) | 0.58 (0.48 to 0.68) | -1.79 (-2.23 to -1.34) | 0.74 (0.67 to 0.82) | 0.32 (0.27 to 0.37) | -3.33 (-3.81 to -2.85) |
| Luxembourg | 4 (3 to 4) | 3 (3 to 4) | -0.04 (-0.14 to 0.07) | 0.94 (0.89 to 0.98) | 0.53 (0.48 to 0.59) | -1.93 (-2.08 to -1.79) | 0.77 (0.73 to 0.81) | 0.36 (0.32 to 0.39) | -2.69 (-2.84 to -2.54) |
| Madagascar | 88 (62 to 137) | 201 (114 to 349) | 1.28 (0.44 to 2.13) | 0.74 (0.52 to 1.15) | 0.7 (0.4 to 1.22) | -0.27 (-0.45 to -0.09) | 1.09 (0.78 to 1.64) | 0.97 (0.58 to 1.64) | -0.4 (-0.51 to -0.28) |
| Malawi | 76 (52 to 111) | 148 (89 to 252) | 0.93 (0.28 to 1.69) | 0.78 (0.53 to 1.13) | 0.76 (0.46 to 1.29) | -0.23 (-0.32 to -0.14) | 1.16 (0.82 to 1.69) | 1.09 (0.69 to 1.83) | -0.28 (-0.36 to -0.2) |
| Malaysia | 56 (31 to 80) | 171 (84 to 260) | 2.07 (1.26 to 3.41) | 0.32 (0.18 to 0.45) | 0.54 (0.26 to 0.82) | 1.77 (1.48 to 2.06) | 0.48 (0.28 to 0.69) | 0.59 (0.29 to 0.89) | 0.61 (0.45 to 0.78) |
| Maldives | 1 (1 to 1) | 2 (2 to 3) | 1.46 (0.68 to 2.47) | 0.41 (0.28 to 0.54) | 0.44 (0.29 to 0.59) | 0.19 (-0.04 to 0.43) | 0.74 (0.51 to 0.97) | 0.58 (0.38 to 0.8) | -0.89 (-1.03 to -0.75) |
| Mali | 38 (27 to 49) | 89 (64 to 116) | 1.34 (0.64 to 2.42) | 0.44 (0.32 to 0.57) | 0.37 (0.27 to 0.48) | -0.49 (-0.57 to -0.42) | 0.6 (0.43 to 0.77) | 0.54 (0.38 to 0.71) | -0.34 (-0.4 to -0.28) |
| Malta | 3 (3 to 4) | 4 (3 to 5) | 0.2 (0.04 to 0.39) | 0.89 (0.81 to 0.98) | 0.89 (0.77 to 1.02) | -0.05 (-0.26 to 0.17) | 0.83 (0.75 to 0.91) | 0.54 (0.47 to 0.62) | -1.49 (-1.71 to -1.26) |
| Marshall Islands | 0 (0 to 0) | 0 (0 to 0) | 1.33 (0.39 to 2.69) | 0.23 (0.07 to 0.48) | 0.44 (0.12 to 0.89) | 1.91 (1.72 to 2.11) | 0.39 (0.13 to 0.8) | 0.55 (0.15 to 1.09) | 1.01 (0.8 to 1.22) |
| Mauritania | 11 (8 to 14) | 21 (14 to 31) | 0.95 (0.25 to 1.88) | 0.51 (0.38 to 0.67) | 0.47 (0.32 to 0.71) | -0.56 (-0.64 to -0.48) | 0.69 (0.51 to 0.89) | 0.63 (0.43 to 0.94) | -0.53 (-0.6 to -0.46) |
| Mauritius | 9 (8 to 9) | 18 (16 to 19) | 0.99 (0.84 to 1.15) | 0.81 (0.77 to 0.85) | 1.39 (1.28 to 1.47) | 3.2 (1.64 to 4.79) | 1.1 (1.04 to 1.16) | 1.01 (0.93 to 1.07) | 1.09 (-0.42 to 2.63) |
| Mexico | 431 (419 to 443) | 1188 (1059 to 1314) | 1.76 (1.46 to 2.07) | 0.5 (0.49 to 0.52) | 0.92 (0.82 to 1.02) | 2.51 (2.01 to 3.01) | 0.79 (0.77 to 0.81) | 0.94 (0.84 to 1.03) | 1.07 (0.58 to 1.55) |
| Micronesia (Federated States of) | 0 (0 to 1) | 1 (0 to 1) | 0.53 (-0.09 to 1.28) | 0.38 (0.15 to 0.68) | 0.59 (0.18 to 1.12) | 1.36 (1.28 to 1.44) | 0.56 (0.24 to 0.99) | 0.69 (0.22 to 1.28) | 0.64 (0.51 to 0.76) |
| Monaco | 0 (0 to 0) | 0 (0 to 0) | 0.18 (-0.19 to 0.81) | 0.15 (0.05 to 0.27) | 0.14 (0.05 to 0.24) | -0.51 (-0.66 to -0.35) | 0.09 (0.03 to 0.16) | 0.08 (0.03 to 0.13) | -0.85 (-1.01 to -0.69) |
| Mongolia | 22 (14 to 32) | 32 (24 to 42) | 0.46 (0.02 to 1.23) | 1.02 (0.66 to 1.47) | 0.97 (0.72 to 1.25) | -0.16 (-0.3 to -0.01) | 1.6 (1.06 to 2.28) | 1.21 (0.91 to 1.56) | -1.04 (-1.19 to -0.9) |
| Montenegro | 5 (3 to 7) | 5 (4 to 7) | 0.01 (-0.32 to 0.47) | 0.77 (0.43 to 1.13) | 0.79 (0.58 to 1.07) | 0.08 (-0.03 to 0.19) | 0.77 (0.43 to 1.12) | 0.57 (0.42 to 0.77) | -1.03 (-1.17 to -0.89) |
| Morocco | 93 (63 to 124) | 186 (124 to 264) | 1 (0.31 to 2.03) | 0.37 (0.25 to 0.49) | 0.5 (0.33 to 0.71) | 1.05 (0.97 to 1.14) | 0.48 (0.34 to 0.66) | 0.53 (0.36 to 0.76) | 0.4 (0.34 to 0.46) |
| Mozambique | 110 (76 to 153) | 250 (145 to 418) | 1.27 (0.42 to 2.29) | 0.82 (0.57 to 1.14) | 0.8 (0.47 to 1.35) | 0.07 (-0.04 to 0.18) | 1.21 (0.84 to 1.66) | 1.22 (0.71 to 2.02) | 0.18 (0.09 to 0.27) |
| Myanmar | 235 (128 to 350) | 509 (230 to 843) | 1.17 (0.27 to 2.72) | 0.58 (0.32 to 0.86) | 0.9 (0.41 to 1.49) | 1.33 (1.22 to 1.44) | 0.8 (0.46 to 1.16) | 1 (0.45 to 1.63) | 0.7 (0.56 to 0.85) |
| Namibia | 10 (7 to 12) | 19 (12 to 27) | 0.93 (0.28 to 1.9) | 0.69 (0.53 to 0.86) | 0.77 (0.5 to 1.13) | 0.13 (-0.15 to 0.41) | 1.09 (0.84 to 1.39) | 1.07 (0.69 to 1.59) | -0.35 (-0.67 to -0.04) |
| Nauru | 0 (0 to 0) | 0 (0 to 0) | 0.45 (-0.1 to 1.19) | 0.39 (0.15 to 0.69) | 0.52 (0.15 to 0.93) | 0.81 (0.65 to 0.98) | 0.59 (0.24 to 0.99) | 0.72 (0.22 to 1.25) | 0.58 (0.44 to 0.72) |
| Nepal | 100 (71 to 136) | 211 (152 to 286) | 1.1 (0.4 to 2.05) | 0.52 (0.36 to 0.7) | 0.68 (0.49 to 0.92) | 1.03 (0.84 to 1.22) | 0.71 (0.51 to 0.94) | 0.76 (0.55 to 1.04) | 0.29 (0.13 to 0.45) |
| Netherlands | 81 (76 to 86) | 87 (77 to 97) | 0.07 (-0.05 to 0.2) | 0.54 (0.51 to 0.58) | 0.5 (0.44 to 0.56) | -0.64 (-0.86 to -0.41) | 0.45 (0.42 to 0.47) | 0.29 (0.25 to 0.32) | -1.9 (-2.16 to -1.64) |
| New Zealand | 27 (25 to 29) | 24 (22 to 27) | -0.1 (-0.21 to 0.02) | 0.79 (0.72 to 0.86) | 0.47 (0.42 to 0.52) | -1.78 (-2.24 to -1.31) | 0.73 (0.67 to 0.79) | 0.37 (0.33 to 0.41) | -2.38 (-2.84 to -1.91) |
| Nicaragua | 16 (12 to 22) | 41 (31 to 53) | 1.53 (0.83 to 2.8) | 0.41 (0.31 to 0.57) | 0.61 (0.46 to 0.79) | 1.52 (1.33 to 1.72) | 0.64 (0.49 to 0.88) | 0.74 (0.56 to 0.97) | 0.73 (0.5 to 0.96) |
| Niger | 34 (24 to 48) | 83 (54 to 121) | 1.42 (0.61 to 2.56) | 0.43 (0.3 to 0.59) | 0.33 (0.22 to 0.49) | -0.94 (-1.11 to -0.76) | 0.62 (0.44 to 0.86) | 0.51 (0.34 to 0.73) | -0.77 (-0.87 to -0.66) |
| Nigeria | 476 (343 to 642) | 1122 (806 to 1507) | 1.36 (0.78 to 2.2) | 0.53 (0.38 to 0.71) | 0.49 (0.35 to 0.65) | -0.3 (-0.4 to -0.2) | 0.71 (0.5 to 0.96) | 0.67 (0.48 to 0.9) | -0.2 (-0.26 to -0.15) |
| Niue | 0 (0 to 0) | 0 (0 to 0) | 0.32 (-0.15 to 1.03) | 0.47 (0.18 to 0.9) | 0.85 (0.28 to 1.63) | 1.47 (1.22 to 1.72) | 0.47 (0.18 to 0.91) | 0.76 (0.26 to 1.44) | 0.88 (0.66 to 1.11) |
| North Macedonia | 34 (28 to 43) | 39 (25 to 59) | 0.13 (-0.27 to 0.54) | 1.73 (1.4 to 2.15) | 1.78 (1.17 to 2.73) | -0.03 (-0.24 to 0.18) | 1.82 (1.48 to 2.25) | 1.33 (0.87 to 2.03) | -1.16 (-1.4 to -0.92) |
| Northern Mariana Islands | 0 (0 to 0) | 0 (0 to 0) | 1.03 (0.19 to 2.31) | 0.22 (0.15 to 0.32) | 0.42 (0.28 to 0.56) | 2.88 (2.39 to 3.36) | 0.37 (0.26 to 0.52) | 0.4 (0.27 to 0.53) | 0.81 (0.35 to 1.26) |
| Norway | 21 (20 to 22) | 31 (28 to 33) | 0.45 (0.35 to 0.58) | 0.5 (0.47 to 0.52) | 0.57 (0.51 to 0.62) | -0.46 (-1.01 to 0.1) | 0.37 (0.35 to 0.39) | 0.4 (0.37 to 0.43) | -0.68 (-1.28 to -0.08) |
| Oman | 2 (1 to 4) | 6 (4 to 8) | 1.36 (0.41 to 2.92) | 0.12 (0.07 to 0.18) | 0.12 (0.08 to 0.16) | 0.29 (0.02 to 0.56) | 0.23 (0.13 to 0.35) | 0.23 (0.16 to 0.31) | 0.17 (-0.01 to 0.36) |
| Pakistan | 672 (519 to 852) | 1849 (1319 to 2627) | 1.75 (0.9 to 2.89) | 0.6 (0.47 to 0.77) | 0.78 (0.56 to 1.12) | 0.72 (0.59 to 0.85) | 0.82 (0.63 to 1.04) | 1.02 (0.73 to 1.43) | 0.57 (0.45 to 0.7) |
| Palau | 0 (0 to 0) | 0 (0 to 0) | 0.5 (-0.02 to 1.3) | 0.06 (0.02 to 0.1) | 0.08 (0.03 to 0.13) | 0.78 (0.61 to 0.96) | 0.08 (0.03 to 0.13) | 0.07 (0.03 to 0.12) | 0 (-0.17 to 0.17) |
| Palestine | 14 (9 to 21) | 38 (29 to 50) | 1.76 (0.98 to 3.41) | 0.67 (0.44 to 1.01) | 0.74 (0.56 to 0.98) | 0.59 (0.37 to 0.81) | 1.09 (0.73 to 1.57) | 1.14 (0.87 to 1.47) | 0.42 (0.27 to 0.56) |
| Panama | 7 (7 to 7) | 30 (24 to 36) | 3.19 (2.38 to 4.05) | 0.3 (0.28 to 0.31) | 0.69 (0.56 to 0.83) | 2.61 (2.26 to 2.97) | 0.39 (0.37 to 0.41) | 0.67 (0.54 to 0.81) | 1.58 (1.22 to 1.94) |
| Papua New Guinea | 9 (2 to 22) | 29 (6 to 70) | 2.19 (1.04 to 3.94) | 0.23 (0.05 to 0.53) | 0.28 (0.06 to 0.67) | 0.64 (0.5 to 0.78) | 0.33 (0.08 to 0.76) | 0.39 (0.08 to 0.93) | 0.5 (0.34 to 0.66) |
| Paraguay | 37 (27 to 47) | 85 (60 to 119) | 1.32 (0.57 to 2.33) | 0.91 (0.68 to 1.17) | 1.19 (0.84 to 1.66) | 1.02 (0.87 to 1.18) | 1.31 (0.96 to 1.66) | 1.37 (0.97 to 1.9) | 0.27 (0.1 to 0.45) |
| Peru | 158 (116 to 203) | 262 (188 to 353) | 0.66 (0.06 to 1.4) | 0.73 (0.53 to 0.94) | 0.72 (0.52 to 0.97) | -0.02 (-0.13 to 0.08) | 0.93 (0.69 to 1.19) | 0.76 (0.54 to 1.02) | -0.85 (-0.99 to -0.71) |
| Philippines | 604 (516 to 800) | 1617 (1267 to 2212) | 1.68 (1.19 to 2.3) | 0.96 (0.82 to 1.27) | 1.43 (1.12 to 1.95) | 1.52 (1.35 to 1.7) | 1.49 (1.26 to 1.99) | 1.77 (1.38 to 2.43) | 0.76 (0.53 to 0.99) |
| Poland | 608 (583 to 632) | 299 (272 to 324) | -0.51 (-0.55 to -0.46) | 1.59 (1.53 to 1.65) | 0.78 (0.71 to 0.85) | -2.57 (-2.82 to -2.32) | 1.46 (1.4 to 1.52) | 0.48 (0.44 to 0.52) | -3.85 (-4.1 to -3.59) |
| Portugal | 162 (152 to 172) | 126 (112 to 140) | -0.22 (-0.3 to -0.13) | 1.6 (1.5 to 1.7) | 1.19 (1.06 to 1.32) | -1.39 (-1.8 to -0.97) | 1.35 (1.26 to 1.42) | 0.67 (0.6 to 0.73) | -2.76 (-3.13 to -2.38) |
| Puerto Rico | 9 (8 to 10) | 25 (21 to 30) | 1.8 (1.28 to 2.43) | 0.25 (0.22 to 0.28) | 0.76 (0.63 to 0.9) | 3.79 (2.43 to 5.16) | 0.25 (0.22 to 0.27) | 0.41 (0.34 to 0.49) | 1.81 (0.4 to 3.24) |
| Qatar | 1 (1 to 2) | 6 (4 to 9) | 4.4 (2.76 to 6.59) | 0.25 (0.17 to 0.35) | 0.21 (0.14 to 0.29) | -0.34 (-0.56 to -0.11) | 0.61 (0.42 to 0.8) | 0.5 (0.33 to 0.71) | -0.42 (-0.55 to -0.28) |
| Republic of Korea | 197 (138 to 255) | 177 (137 to 233) | -0.1 (-0.37 to 0.48) | 0.45 (0.31 to 0.58) | 0.34 (0.27 to 0.45) | -1.1 (-1.22 to -0.98) | 0.52 (0.37 to 0.65) | 0.25 (0.19 to 0.33) | -2.7 (-2.84 to -2.56) |
| Republic of Moldova | 66 (58 to 74) | 28 (25 to 32) | -0.58 (-0.65 to -0.48) | 1.49 (1.31 to 1.67) | 0.78 (0.69 to 0.89) | -2.54 (-2.88 to -2.2) | 1.51 (1.33 to 1.68) | 0.51 (0.45 to 0.58) | -3.93 (-4.28 to -3.58) |
| Romania | 497 (403 to 596) | 310 (258 to 367) | -0.38 (-0.5 to -0.21) | 2.12 (1.72 to 2.55) | 1.64 (1.36 to 1.94) | -1.05 (-1.19 to -0.92) | 1.87 (1.53 to 2.22) | 0.97 (0.81 to 1.14) | -2.38 (-2.53 to -2.22) |
| Russian Federation | 3448 (3321 to 3544) | 977 (900 to 1052) | -0.72 (-0.74 to -0.69) | 2.28 (2.2 to 2.35) | 0.67 (0.62 to 0.73) | -5.07 (-5.43 to -4.71) | 2.02 (1.95 to 2.08) | 0.5 (0.46 to 0.53) | -5.54 (-5.87 to -5.2) |
| Rwanda | 71 (48 to 107) | 106 (58 to 192) | 0.49 (-0.11 to 1.37) | 0.99 (0.67 to 1.48) | 0.8 (0.43 to 1.44) | -1.08 (-1.26 to -0.91) | 1.44 (1.03 to 2.09) | 1.08 (0.61 to 1.94) | -1.32 (-1.47 to -1.16) |
| Saint Kitts and Nevis | 0 (0 to 0) | 0 (0 to 1) | 0.01 (-0.16 to 0.21) | 1.08 (0.97 to 1.17) | 0.77 (0.64 to 0.9) | -0.91 (-1.34 to -0.47) | 1.15 (1.03 to 1.24) | 0.74 (0.62 to 0.85) | -1.12 (-1.45 to -0.78) |
| Saint Lucia | 1 (1 to 1) | 1 (1 to 1) | 0.57 (0.28 to 0.87) | 0.53 (0.49 to 0.58) | 0.64 (0.53 to 0.77) | 0.68 (0.47 to 0.89) | 0.72 (0.67 to 0.78) | 0.52 (0.43 to 0.62) | -1.12 (-1.28 to -0.96) |
| Saint Vincent and the Grenadines | 0 (0 to 0) | 2 (2 to 2) | 4.36 (3.6 to 5.2) | 0.36 (0.33 to 0.39) | 1.86 (1.63 to 2.12) | 4.81 (3.55 to 6.09) | 0.46 (0.42 to 0.5) | 1.61 (1.41 to 1.83) | 3.47 (2.18 to 4.78) |
| Samoa | 1 (1 to 3) | 2 (1 to 4) | 0.55 (0.14 to 1.75) | 0.86 (0.45 to 1.54) | 1.06 (0.64 to 1.73) | 0.84 (0.69 to 1) | 1.22 (0.64 to 2.19) | 1.33 (0.82 to 2.15) | 0.42 (0.28 to 0.56) |
| San Marino | 0 (0 to 0) | 0 (0 to 0) | -0.05 (-0.39 to 0.38) | 0.86 (0.6 to 1.22) | 0.6 (0.36 to 0.94) | -0.55 (-0.86 to -0.24) | 0.65 (0.46 to 0.91) | 0.33 (0.21 to 0.52) | -1.47 (-1.73 to -1.2) |
| Sao Tome and Principe | 1 (0 to 1) | 1 (1 to 1) | 0.87 (0.1 to 2.06) | 0.43 (0.29 to 0.67) | 0.45 (0.3 to 0.68) | 0.01 (-0.13 to 0.15) | 0.53 (0.36 to 0.81) | 0.57 (0.39 to 0.82) | 0.13 (0.02 to 0.25) |
| Saudi Arabia | 71 (48 to 98) | 194 (138 to 272) | 1.74 (0.8 to 3.22) | 0.45 (0.3 to 0.62) | 0.51 (0.37 to 0.72) | 0.44 (0.39 to 0.49) | 0.74 (0.51 to 1.02) | 0.75 (0.56 to 1.03) | 0.03 (-0.05 to 0.1) |
| Senegal | 37 (27 to 53) | 76 (51 to 109) | 1.06 (0.35 to 1.91) | 0.48 (0.35 to 0.7) | 0.48 (0.32 to 0.69) | 0.08 (-0.07 to 0.23) | 0.65 (0.47 to 0.94) | 0.62 (0.42 to 0.86) | -0.1 (-0.22 to 0.02) |
| Serbia | 142 (88 to 219) | 128 (95 to 170) | -0.1 (-0.47 to 0.42) | 1.48 (0.91 to 2.28) | 1.44 (1.07 to 1.91) | -0.24 (-0.33 to -0.16) | 1.39 (0.84 to 2.09) | 0.87 (0.64 to 1.14) | -1.75 (-1.84 to -1.65) |
| Seychelles | 0 (0 to 0) | 0 (0 to 0) | 0.61 (0.19 to 1.17) | 0.18 (0.05 to 0.31) | 0.2 (0.05 to 0.35) | 0.72 (0.17 to 1.27) | 0.22 (0.06 to 0.36) | 0.19 (0.05 to 0.32) | -0.03 (-0.51 to 0.45) |
| Sierra Leone | 16 (11 to 24) | 34 (23 to 49) | 1.09 (0.41 to 1.98) | 0.39 (0.26 to 0.58) | 0.38 (0.26 to 0.55) | -0.04 (-0.18 to 0.1) | 0.51 (0.34 to 0.76) | 0.5 (0.34 to 0.72) | 0 (-0.1 to 0.1) |
| Singapore | 12 (11 to 13) | 15 (13 to 17) | 0.18 (0.03 to 0.36) | 0.41 (0.37 to 0.44) | 0.26 (0.22 to 0.29) | -1.77 (-2.04 to -1.5) | 0.47 (0.43 to 0.5) | 0.23 (0.2 to 0.26) | -2.6 (-2.91 to -2.29) |
| Slovakia | 50 (36 to 68) | 49 (34 to 67) | -0.02 (-0.35 to 0.38) | 0.94 (0.69 to 1.29) | 0.9 (0.63 to 1.24) | -0.24 (-0.3 to -0.17) | 0.86 (0.63 to 1.19) | 0.58 (0.42 to 0.8) | -1.31 (-1.36 to -1.25) |
| Slovenia | 13 (12 to 15) | 11 (9 to 13) | -0.17 (-0.32 to 0.01) | 0.68 (0.61 to 0.74) | 0.54 (0.44 to 0.64) | -0.92 (-1.35 to -0.49) | 0.57 (0.52 to 0.63) | 0.28 (0.23 to 0.33) | -2.55 (-2.99 to -2.11) |
| Solomon Islands | 1 (0 to 2) | 3 (1 to 5) | 2.09 (1.09 to 3.96) | 0.24 (0.06 to 0.57) | 0.37 (0.09 to 0.79) | 1.25 (1.12 to 1.37) | 0.39 (0.1 to 0.9) | 0.52 (0.13 to 1.11) | 0.85 (0.72 to 0.97) |
| Somalia | 56 (36 to 87) | 139 (75 to 236) | 1.49 (0.54 to 2.62) | 0.7 (0.45 to 1.09) | 0.64 (0.35 to 1.09) | -0.49 (-0.65 to -0.34) | 1.24 (0.81 to 1.88) | 1.1 (0.63 to 1.84) | -0.4 (-0.47 to -0.33) |
| South Africa | 176 (115 to 220) | 285 (235 to 359) | 0.62 (0.25 to 1.33) | 0.47 (0.31 to 0.59) | 0.5 (0.41 to 0.63) | -0.15 (-0.44 to 0.14) | 0.66 (0.42 to 0.83) | 0.58 (0.47 to 0.71) | -0.78 (-1.08 to -0.49) |
| South Sudan | 45 (29 to 75) | 80 (43 to 148) | 0.77 (0.05 to 1.63) | 0.77 (0.5 to 1.28) | 0.83 (0.45 to 1.53) | 0 (-0.29 to 0.3) | 1.19 (0.79 to 1.91) | 1.23 (0.71 to 2.24) | 0.03 (-0.08 to 0.15) |
| Spain | 456 (429 to 479) | 362 (322 to 399) | -0.21 (-0.28 to -0.13) | 1.17 (1.11 to 1.23) | 0.79 (0.71 to 0.88) | -1.29 (-1.57 to -1) | 0.94 (0.89 to 0.98) | 0.48 (0.43 to 0.53) | -2.07 (-2.29 to -1.86) |
| Sri Lanka | 51 (40 to 68) | 144 (73 to 242) | 1.83 (0.36 to 4.1) | 0.3 (0.23 to 0.4) | 0.65 (0.33 to 1.08) | 3.3 (2.89 to 3.71) | 0.4 (0.31 to 0.54) | 0.56 (0.28 to 0.91) | 1.88 (1.44 to 2.32) |
| Sudan | 94 (54 to 139) | 224 (142 to 336) | 1.39 (0.45 to 2.87) | 0.47 (0.27 to 0.7) | 0.51 (0.33 to 0.77) | 0.36 (0.26 to 0.45) | 0.67 (0.41 to 0.97) | 0.73 (0.49 to 1.04) | 0.32 (0.25 to 0.39) |
| Suriname | 2 (2 to 3) | 5 (3 to 6) | 0.93 (0.32 to 1.92) | 0.62 (0.47 to 0.82) | 0.8 (0.57 to 1.1) | 0.97 (0.83 to 1.11) | 0.8 (0.6 to 1.04) | 0.76 (0.54 to 1.04) | 0 (-0.08 to 0.08) |
| Sweden | 64 (59 to 68) | 39 (35 to 44) | -0.38 (-0.46 to -0.3) | 0.74 (0.69 to 0.79) | 0.38 (0.33 to 0.43) | -1.85 (-2.12 to -1.57) | 0.55 (0.51 to 0.59) | 0.25 (0.22 to 0.28) | -2.33 (-2.65 to -2.01) |
| Switzerland | 36 (34 to 39) | 34 (29 to 38) | -0.07 (-0.19 to 0.07) | 0.53 (0.49 to 0.57) | 0.38 (0.33 to 0.43) | -1.35 (-1.55 to -1.14) | 0.41 (0.38 to 0.44) | 0.22 (0.19 to 0.25) | -2.33 (-2.56 to -2.09) |
| Syrian Arab Republic | 19 (10 to 29) | 30 (16 to 47) | 0.6 (0.05 to 1.55) | 0.15 (0.08 to 0.23) | 0.21 (0.11 to 0.34) | 0.91 (0.53 to 1.3) | 0.23 (0.12 to 0.35) | 0.23 (0.12 to 0.35) | -0.17 (-0.24 to -0.09) |
| Taiwan (Province of China) | 147 (137 to 156) | 148 (132 to 165) | 0.01 (-0.09 to 0.13) | 0.72 (0.67 to 0.76) | 0.63 (0.56 to 0.7) | 0.01 (-0.49 to 0.51) | 0.86 (0.8 to 0.91) | 0.4 (0.36 to 0.44) | -2.03 (-2.49 to -1.56) |
| Tajikistan | 52 (36 to 72) | 93 (52 to 139) | 0.78 (-0.08 to 1.55) | 0.98 (0.67 to 1.33) | 0.92 (0.51 to 1.37) | -0.38 (-0.49 to -0.28) | 1.51 (1.04 to 2.06) | 1.21 (0.71 to 1.77) | -1 (-1.12 to -0.88) |
| Thailand | 384 (303 to 486) | 1185 (753 to 1651) | 2.09 (0.99 to 3.72) | 0.68 (0.53 to 0.86) | 1.78 (1.13 to 2.48) | 3.6 (3.46 to 3.74) | 0.87 (0.68 to 1.14) | 1.22 (0.78 to 1.69) | 1.49 (1.32 to 1.66) |
| Timor-Leste | 3 (2 to 4) | 9 (4 to 14) | 2.17 (0.94 to 3.91) | 0.36 (0.2 to 0.52) | 0.64 (0.28 to 1.04) | 2.18 (2 to 2.36) | 0.68 (0.39 to 0.95) | 0.88 (0.38 to 1.42) | 1 (0.89 to 1.11) |
| Togo | 15 (10 to 21) | 34 (22 to 51) | 1.33 (0.56 to 2.37) | 0.4 (0.28 to 0.59) | 0.41 (0.27 to 0.61) | -0.06 (-0.13 to 0.02) | 0.58 (0.41 to 0.85) | 0.55 (0.37 to 0.81) | -0.29 (-0.35 to -0.24) |
| Tokelau | 0 (0 to 0) | 0 (0 to 0) | 0.74 (0.15 to 1.67) | 0.42 (0.15 to 0.86) | 0.86 (0.27 to 1.63) | 1.58 (1.26 to 1.9) | 0.47 (0.17 to 0.95) | 0.81 (0.26 to 1.53) | 0.91 (0.6 to 1.23) |
| Tonga | 0 (0 to 0) | 0 (0 to 1) | 0.76 (0.01 to 1.58) | 0.28 (0.12 to 0.48) | 0.45 (0.14 to 0.89) | 1.47 (1.26 to 1.69) | 0.38 (0.16 to 0.68) | 0.53 (0.16 to 1.04) | 0.98 (0.78 to 1.18) |
| Trinidad and Tobago | 8 (8 to 9) | 11 (8 to 14) | 0.38 (0.04 to 0.76) | 0.67 (0.62 to 0.71) | 0.79 (0.61 to 1) | 0.57 (0.4 to 0.74) | 0.85 (0.8 to 0.9) | 0.64 (0.49 to 0.8) | -0.93 (-1.1 to -0.75) |
| Tunisia | 41 (31 to 52) | 79 (54 to 114) | 0.94 (0.26 to 1.89) | 0.49 (0.37 to 0.62) | 0.66 (0.45 to 0.97) | 1.01 (0.97 to 1.04) | 0.64 (0.49 to 0.84) | 0.64 (0.44 to 0.92) | 0.02 (-0.02 to 0.05) |
| Turkmenistan | 38 (27 to 50) | 53 (34 to 79) | 0.42 (-0.13 to 1.34) | 1.02 (0.73 to 1.35) | 1.04 (0.65 to 1.54) | -0.18 (-0.26 to -0.09) | 1.54 (1.09 to 2.09) | 1.2 (0.76 to 1.78) | -1.1 (-1.22 to -0.97) |
| Tuvalu | 0 (0 to 0) | 0 (0 to 0) | 0.83 (0.14 to 1.61) | 0.36 (0.12 to 0.71) | 0.5 (0.14 to 0.99) | 0.95 (0.78 to 1.12) | 0.45 (0.16 to 0.9) | 0.55 (0.15 to 1.08) | 0.55 (0.43 to 0.67) |
| Uganda | 135 (98 to 178) | 362 (242 to 552) | 1.69 (0.66 to 3.31) | 0.78 (0.56 to 1.03) | 0.84 (0.56 to 1.27) | -0.42 (-0.67 to -0.17) | 1.25 (0.92 to 1.62) | 1.25 (0.86 to 1.92) | -0.57 (-0.81 to -0.34) |
| Ukraine | 408 (363 to 453) | 349 (255 to 454) | -0.14 (-0.38 to 0.14) | 0.77 (0.69 to 0.86) | 0.81 (0.59 to 1.05) | -0.16 (-0.42 to 0.09) | 0.65 (0.58 to 0.72) | 0.6 (0.44 to 0.78) | -0.54 (-0.74 to -0.34) |
| United Arab Emirates | 7 (5 to 9) | 29 (22 to 38) | 3.2 (1.91 to 4.6) | 0.37 (0.26 to 0.49) | 0.3 (0.22 to 0.4) | -0.54 (-0.71 to -0.36) | 0.87 (0.6 to 1.18) | 0.7 (0.48 to 0.94) | 0.77 (0.33 to 1.21) |
| United Kingdom | 333 (323 to 340) | 445 (417 to 462) | 0.34 (0.28 to 0.38) | 0.58 (0.56 to 0.59) | 0.66 (0.61 to 0.68) | 0.52 (0.25 to 0.78) | 0.47 (0.46 to 0.48) | 0.46 (0.44 to 0.48) | -0.02 (-0.26 to 0.22) |
| United Republic of Tanzania | 229 (160 to 337) | 481 (271 to 851) | 1.1 (0.28 to 1.95) | 0.89 (0.62 to 1.3) | 0.82 (0.46 to 1.46) | -0.26 (-0.34 to -0.17) | 1.29 (0.94 to 1.86) | 1.14 (0.68 to 2) | -0.44 (-0.49 to -0.39) |
| United States of America | 1254 (1200 to 1288) | 2094 (1939 to 2183) | 0.67 (0.61 to 0.72) | 0.49 (0.47 to 0.51) | 0.63 (0.58 to 0.66) | 0.62 (0.43 to 0.81) | 0.43 (0.42 to 0.45) | 0.45 (0.43 to 0.47) | -0.02 (-0.15 to 0.11) |
| United States Virgin Islands | 0 (0 to 0) | 0 (0 to 0) | 0.45 (-0.08 to 1.3) | 0.2 (0.13 to 0.28) | 0.36 (0.18 to 0.57) | 2.46 (2.31 to 2.61) | 0.24 (0.16 to 0.33) | 0.2 (0.1 to 0.33) | 0.09 (-0.09 to 0.27) |
| Uruguay | 8 (7 to 9) | 8 (7 to 9) | -0.06 (-0.2 to 0.11) | 0.27 (0.23 to 0.3) | 0.23 (0.21 to 0.26) | -0.59 (-0.96 to -0.21) | 0.23 (0.2 to 0.26) | 0.16 (0.14 to 0.18) | -1.25 (-1.63 to -0.87) |
| Uzbekistan | 138 (104 to 175) | 325 (261 to 396) | 1.36 (0.68 to 2.47) | 0.66 (0.5 to 0.84) | 0.95 (0.76 to 1.16) | 1.55 (1.11 to 1.99) | 0.97 (0.69 to 1.27) | 1.11 (0.88 to 1.35) | 0.71 (0.28 to 1.15) |
| Vanuatu | 0 (0 to 1) | 1 (0 to 2) | 2.4 (1.32 to 3.99) | 0.23 (0.07 to 0.49) | 0.38 (0.1 to 0.76) | 1.45 (1.31 to 1.6) | 0.37 (0.12 to 0.79) | 0.52 (0.14 to 1.05) | 0.97 (0.83 to 1.11) |
| Venezuela (Bolivarian Republic of) | 117 (106 to 127) | 284 (216 to 362) | 1.43 (0.79 to 2.21) | 0.62 (0.56 to 0.68) | 1.07 (0.81 to 1.36) | 1.87 (1.59 to 2.16) | 0.93 (0.84 to 1) | 1.01 (0.77 to 1.28) | 0.46 (0.23 to 0.69) |
| Viet Nam | 423 (314 to 563) | 986 (714 to 1298) | 1.33 (0.69 to 2.32) | 0.62 (0.46 to 0.83) | 0.98 (0.71 to 1.29) | 1.57 (1.48 to 1.66) | 0.87 (0.63 to 1.18) | 1.01 (0.74 to 1.32) | 0.53 (0.43 to 0.63) |
| Yemen | 38 (18 to 61) | 112 (57 to 190) | 1.97 (0.91 to 3.65) | 0.28 (0.13 to 0.45) | 0.33 (0.17 to 0.57) | 0.54 (0.43 to 0.66) | 0.52 (0.28 to 0.84) | 0.56 (0.3 to 0.9) | 0.23 (0.16 to 0.3) |
| Zambia | 69 (49 to 103) | 150 (83 to 266) | 1.19 (0.31 to 2.27) | 0.86 (0.61 to 1.29) | 0.77 (0.43 to 1.36) | -0.52 (-0.61 to -0.43) | 1.3 (0.94 to 1.87) | 1.15 (0.66 to 2.02) | -0.53 (-0.61 to -0.45) |
| Zimbabwe | 53 (39 to 69) | 103 (70 to 157) | 0.96 (0.25 to 1.91) | 0.51 (0.38 to 0.67) | 0.66 (0.45 to 1.01) | 0.83 (0.4 to 1.26) | 0.93 (0.68 to 1.26) | 1.04 (0.73 to 1.59) | 0.46 (0.07 to 0.85) |

CR, crude rate; ASR, age-standardized rate; EAPC, estimated annual percentage change; UI, uncertainty interval; CI, confidence interval.
